# Efficient pecG-*n* (*n* = 1, 2) Basis Sets for Ga, Ge, As, Se, and Br Specialized for the Geometry Optimization of Molecular Structures

**DOI:** 10.3390/ijms26178197

**Published:** 2025-08-23

**Authors:** Yuriy Yu. Rusakov, Irina L. Rusakova

**Affiliations:** A. E. Favorsky Irkutsk Institute of Chemistry, Siberian Branch of the Russian Academy of Sciences, Favorsky St. 1, 664033 Irkutsk, Russia; rusakov82@mail.ru

**Keywords:** pecG-1, pecG-2, basis set, PEC method, equilibrium geometry, gallium, germanium, arsenic, selenium, bromine

## Abstract

In this paper, efficient pecG-*n* (*n* = 1, 2) basis sets for the 4th period p-elements, Ga, Ge, As, Se, and Br, specified for the optimization of molecular structures, are proposed. These basis sets were optimized via the property-energy consistent (PEC) algorithm directed to the minimization of molecular energy gradient relative to the bond lengths. The performance of the presented basis sets was tested against both theoretical and gas phase electron diffraction experimental reference data relative to the other popular basis sets that are usually employed for the geometry optimization of molecular structures. It was shown that the pecG-*n* (*n* = 1, 2) basis sets give equilibrium molecular structures of the quality that considerably surpasses the quality provided by the other commensurate basis sets.

## 1. Introduction

The quality of molecular equilibrium geometries is of crucial importance in quantum chemistry, as it usually represents a starting point for the calculation of any molecular property. At that, even a small alternation of the molecular equilibrium geometry, for example, a change in the bond lengths in thousandths of Å, can substantially affect different physicochemical properties calculated upon it [1,2]. Reducing the geometry factor error is very important for compounds containing 4th period p-elements, because these molecules have many areas of application in modern technology and an improper prediction of their properties, partially due to the geometry factor error, may substantially distort the knowledge about their usefulness in these areas. In particular, gallium, germanium, and arsenic are known to form efficient semiconductor materials [3,4,5,6,7,8,9,10], serving, for example, as quantum dots [11,12,13,14] with particular electronic and optical properties. Selenium and bromine compounds are of more interest in medicine and drug design [15,16,17,18,19,20,21], though, the use of selenium compounds gradually becomes more and more proliferated in many other areas, such as photoelectronics [22,23], solar cells technology [24], semiconductors industry [25,26], nanotechnology [27,28,29], synthesis of channelized porous materials [30,31], and some other.

At any level of electronic theory, the quality of the equilibrium geometry of a molecule can be substantially improved by increasing the basis set quality [32,33,34,35]. In common terms, this implies an increase in the cardinal number and extending the basis set functional space. In the case of 4th period atoms, such a basis set extension can be very significant as compared to the 1–3 period atoms, due to the fact that electrons of the 4th period elements occupy higher energy levels and have a greater spatial extent, requiring larger basis sets to accurately represent their behavior. Thus, the efficient basis sets for 4th period atoms that are capable of giving accurate results are of great interest in modern quantum chemical calculations.

There are many energy-optimized basis sets of different types and quality, including popular basis set series of Dunning [36], Jensen [37], People [38], Ahlrichs [39], that are used in the geometry optimization of molecules. The main criterion that was used in the optimization of such basis sets is the molecular or atomic energy minimum principle. Thus, these basis sets are naturally suitable for the energy calculations, and are surely applicable to the optimization of geometric parameters. However, the efficacy of the geometry optimization can substantially be increased by using specific exponents, optimized in such a way that a smaller number of them is required in order to obtain geometrical parameters of the same quality as those provided by large standard energy-optimized basis sets. Last year, we proposed a new approach to designing such exponents [1,2]. The idea consisted of using the property–energy consistent (PEC) algorithm [40] modified so as to minimize molecular energy gradient relative to the bond lengths consistent with the molecular energy minimization. Putting this idea into practice, we have already generated a series of the so-called geometry-oriented pecG-*n* (*n* = 1, 2) basis sets for hydrogen and p-elements of 2–3 period atoms [1,2]. These pecG-*n* basis sets turned out to be very effective in the calculation of equilibrium geometries of various compounds with atoms of 1–3 periods, giving geometrical parameters of a quality which is comparable with that provided by considerably larger energy-optimized basis sets.

Considering the utmost importance of the efficiency issue in predicting equilibrium geometries of compounds with 4th period p-elements and already proven usefulness of the pecG-*n* (*n* = 1, 2) basis sets for 1–3 period atoms, we propose herewith the application of our geometry-modified PEC approach to generating the geometry-oriented pecG-*n* (*n* = 1, 2) basis sets for Ga, Ge, As, Se, and Br. In the case of 4th period elements, the creation of basis sets by means of the PEC method is a much more demanding procedure than for the elements of previous periods, requiring, in particular, a more cautious approach to choosing fitting molecules and the usage of a modified algorithm adopted for dealing with large functional spaces. In this work, we have restricted the creation of the pecG-*n* basis set for 4th period atoms by considering particular five p-elements of the most active interest in many applications, while the development of the basis sets for d-elements of 4th period would require a considerably modified PEC approach due to their essentially different valence shell structure, therefore, this represents a separate demanding task worth accomplishing in the nearest future.

## 2. Results

The PEC approach is based on the property-energy consistent optimization of exponents for the chosen target property. In the case of geometry-oriented basis sets, the target property is the molecular energy gradient relative to the bond lengths of the selected bonds that involve a particular atom. In this respect, the PEC optimization represents finding a set of exponents that provide equilibrium bond lengths as close to the corresponding ideal equilibrium values as possible, under the condition that they give the lowest molecular energy. This can be expressed by Equations (1) and (2) as follows:

(1) $$\tilde{\Delta }=\frac{1}{N_{bond}}\sum_{i=1}^{N_{bond}}\left|{\tilde{L}}_{i}-L_{i}^{ideal}\right|\to min$$

(2) $${\tilde{E}}_{tot}=\frac{1}{N_{bond}}\sum_{i=1}^{N_{mol}}{\tilde{E}}_{i}\to min$$

In Equation (1), the $\tilde{\Delta }$ represents a varying target function that is the mean absolute deviation of the $N_{bond}$ varying bond lengths (${\tilde{L}}_{i}$) in the $N_{mol}$ fitting molecules relative to the corresponding ideal bond lengths ($L_{i}^{ideal}$). Equation (2) introduces the energy-minimization principle that guarantees that found exponents not only provide the bond lengths within a desired range but also give the lowest possible energy at that. In general, the PEC algorithm can be thought of as a nonlinear Monte-Carlo-based optimization problem with multiple solutions that can be pictured as random walking on the surface of multidimensional exponential space aimed at finding an isoline of “ideal” values of bond lengths. All points (basis sets) of this isoline give the same “ideal” values, but different total molecular energies. Among those energies, the PEC method selects the basis set that provides the lowest of them. For more details, please, see our main paper on the PEC method [40], and all other, including those pertaining geometry-oriented basis sets [1,2] and the basis sets developed for different NMR properties [41,42,43,44,45,46].

In generating the pecG-*n* basis sets for the 4th period elements, we have used several fitting molecules per atom: CH_3_GaH_2_, Ga_2_H_2_, and Ga_2_H_4_ for Ga; Ge_2_H_6_, Ge_2_H_4_, and GeH_3_CH_3_ for Ge; As_2_H_4_, As_2_H_2_ and H_2_AsCH_3_ for As; Se_2_H_2_ and HSeCH_3_ for Se; Br_2_ and CH_3_Br for Br. The uncontracted structures of the first- and second-level pecG-*n* basis sets were chosen so as to be exactly the same as structures of Dunning’s basis sets for the 4th period elements of double- and triple-zeta quality: [36] (14s,11p,6d) and (20s,13p,9d,1f), respectively. The PEC optimization of the exponents for each atom was carried out resorting to the optimization of geometric structures of corresponding fitting molecules performed within the DFT(B97-2) [47,48] method. During the basis sets optimization, previously obtained pecG-*n* (*n* = 1, 2) basis sets in contracted form were used on hydrogen and carbon atoms.

The “ideal” equilibrium bond lengths were obtained at the DFT(B97-2)/cc-pV5Z level of theory. The cc-pV5Z basis set represents the basis set of the highest cardinal number for the 4th period atoms in the cc-pVXZ (X = D, T, Q, 5) hierarchy [36]. This basis set is very large in size (specifically for the 4th period atoms, it contains 104 basis functions and bears the following structure: [8s,7p,5d,3f,2g,1h]), which makes it a good reference point in any equilibrium geometry calculation. In particular, it was shown recently that the mean absolute percentage error (MAPE), estimated for the equilibrium bond lengths calculated at the DFT(B97-2) level with the cc-pV5Z basis set in a wide variety of molecules containing 1-3 period atoms, relative to the corresponding values obtained with the cc-pV6Z basis set, equals only to 0.01 % [2]. This indicates a negligible difference between the cc-pV5Z and cc-pV6Z basis sets and allows us to consider the cc-pV5Z basis set as the one giving practically meaningful converged values of equilibrium geometry parameters within the cc-pVXZ hierarchy. In addition, in the mentioned paper, we also showed that the B97-2 functional provides the smallest deviations from highly accurate coupled clusters singles and doubles model (CCSD) geometries when the cc-pV5Z basis set is used.

The obtained uncontracted pecG-*n* basis sets were contracted using the PEC method and general contraction scheme [49]. The contraction coefficients were varied so as to minimize the function $\tilde{\Delta }$ (see Equation (1)) relative to the target bond lengths calculated with the uncontracted (for particular shells) pecG-*n* basis sets, under the energetic constraint (see Equation (2)). For more details, please see the contraction of the pecG-n basis sets for atoms of previous periods [1,2]. The final contracted structures of the pecG-1 and pecG-2 basis sets are the same as those of the cc-pVDZ and cc-pVTZ basis sets, being as follows: [5s,4p,2d] and [6s,5p,3d,1f], respectively.

The exponents and contraction coefficients for the pecG-*n* (*n* = 1, 2) basis sets are presented in the Supplementary Materials in Gaussian format.

## 3. Discussion

### 3.1. Theoretical Testing of the pecG-n (n = 1, 2) Basis Sets on the Equilibrium Bond Lengths

In this section, the performance of new pecG-*n* (*n* = 1, 2) basis sets was tested on the example of equilibrium bond lengths of 18 molecules via theoretical analysis. These molecules are as follows: As_4_ (**1**), AsBr_3_ (**2**), AsCl_3_ (**3**), AsF_3_ (**4**), CHBr_3_ (**5**), GaBr_3_ (**6**), GaCl_3_ (**7**), GeBr_2_ (**8**), O(GeH_3_)_2_ (**9**), PF_2_HSe (**10**), S(GeH_3_)_2_ (**11**), SeBr_2_ (**12**), SeCl_2_ (**13**), Se(SiH_3_)_2_ (**14**), Br_2_ (**15**), Me_2_GeF_2_ (**16**), AsP_3_ (17), HCBrClF (**18**).

Theoretical analysis has been performed within the DFT method with the B97-2 exchange-correlation functional that was used for generating the pecG-*n* basis sets, and within the second-order Møller–Plesset perturbation theory MP2 [50,51], which represents an ab initio method typically accounting for 80–90% of the correlation energy [52]. The performance of the pecG-*n* basis sets was compared to that of the Jensen’s pc-*n* (*n* = 1, 2) [37], Pople’s 6-31G(d,p) [53] and 6-311G(d,p) [38], and Dunning’s cc-pVXZ (X = D, T) basis sets [36], being of commensurate sizes with each other at both double- and triple-zeta levels of valence splitting. Please, note that whenever the calculations involved the pecG-*n* (*n* = 1, 2) basis sets, new pecG-*n* were assumed for the 4th period elements, while the rest of the atoms in the molecule were described with the previously proposed pecG-*n* (*n* = 1, 2) basis sets [1,2].

The reference theoretical values of equilibrium bond lengths have been calculated within each of the two methods of electronic theory using the cc-pV5Z basis, that is large enough ([8s,7p,5d,3f,2g,1h] for 4th period, 104 basis functions) to provide a nearly complete basis set limit (CBS). All values of equilibrium bond lengths calculated at the DFT(B97-2) and MP2 levels of theory with different basis sets are given in Tables S1 and S2.

**Figure 1:**
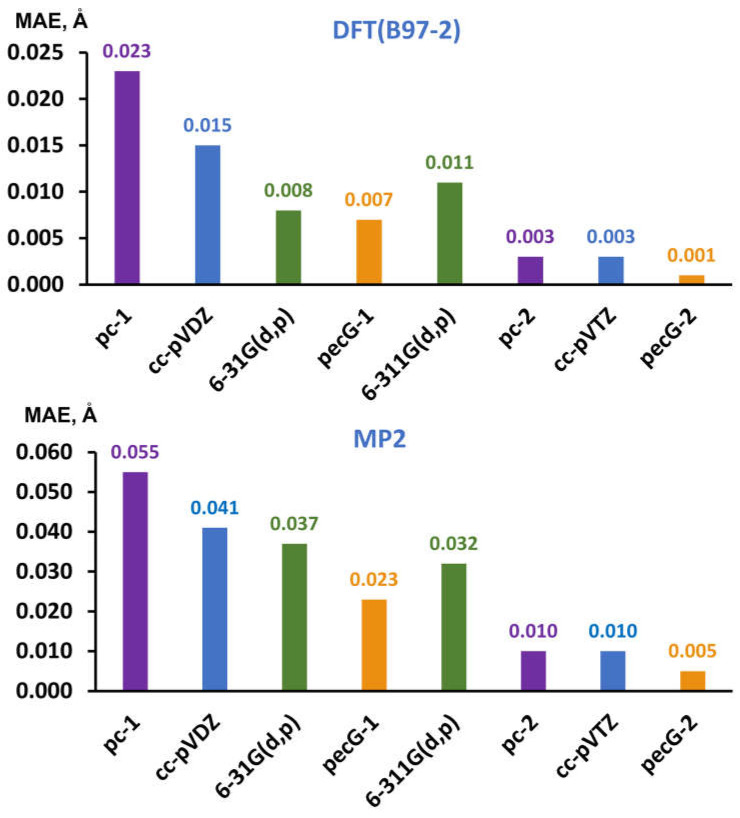
MAEs (in Å) for the equilibrium bond lengths in molecules **1**–**18** obtained in the optimization of their geometrical structures performed within the DFT(B97-2) (upper diagram) and MP2 (bottom diagram) levels of theory using different basis sets, evaluated against corresponding theoretical reference data. All calculated equilibrium bond lengths that were used to estimate the MAEs depicted in this Figure can be found in Tables S1 and S2 in Supplementary Materials.

In order to statistically estimate the measure of deviation of values of equilibrium bond lengths in molecules **1**–**18** calculated with different basis sets against the corresponding theoretical reference data, we have evaluated the mean absolute errors (MAEs, in Å) for the results obtained with each of the basis sets under consideration. The obtained MAEs are presented in Figure 1.

As can be seen from Figure 1, our new pecG-*n* (*n* = 1, 2) basis sets show the best accuracy for both cardinal numbers within all considered levels of electronic theory. Namely, for the compounds containing 4th period atoms, the pecG-*n* (*n* = 1, 2) basis sets provide equilibrium bond lengths, which are up to three and two times more accurate as compared to those obtained with the commensurate basis sets at the DFT and MP2 levels, respectively. At the same time, it is interesting to note that previously introduced pecG-*n* (*n* = 1, 2) basis sets for 1–3 periods [1,2] demonstrated a superior accuracy, which was characterized by six and two times less error for equilibrium bond lengths calculated at the DFT and CCSD levels.

It is also worth mentioning that the 6-31G(d,p) basis set demonstrates the second-best performance among the first-level basis sets, even outperforming the 6-311G(d,p) basis set at the DFT(B97-2) level and closely approaching it at the MP2 level.

We have also checked the valence angles in compounds **1**–**18** (see Tables S3 and S4) and found that the alternations in MAEs for bond angles in going from one basis set to another are insignificant. To be more precise, for the first-level basis sets (including pecG-1), the MAE for angles varies in the range of ca. 0.2–0.3° and 0.7–0.8° for the DFT(B97-2) and MP2 method, respectively. For the optimizations with the second-level basis sets (including pecG-2), these ranges diapasons are noticeably less, namely, around 0.05° and 0.5–0.7° for the DFT(B97-2) and MP2 method, respectively. It is also worth noting that the MAEs for the angles obtained in the optimizations with the 6-311G(d,p), performed within both considered methods, turned out to be among the MAEs for the first-level basis sets.

### 3.2. Testing the Performance of the pecG-n (n = 1, 2) Basis Sets by Comparison of the Calculated Bond Lengths with the Gas Phase Electron Diffraction Experiment

The other test that we have carried out for the pecG-*n* (*n* = 1, 2) basis sets is based on the comparison of theoretical values of bond lengths in molecules **1**–**18** with available gas phase electron diffraction (GED) experiment [54,55,56,57,58,59,60,61,62,63,64,65,66,67,68,69]. Experimental GED data provides an effective internuclear distance bearing a degree of vibrational averaging with taking into account the effect of anharmonicity in the bond-stretching vibrations.

We have estimated the effective internuclear distances *r*_eff_, which may be either *r*_g_ or *r*_a_ determined in the sense given by Bartell [70], depending on the available experimental data. The basic values were calculated as the equilibrium bond lengths *r*_e_ within the DFT(B97-2) or coupled clusters singles and doubles model with perturbative correction for triple excitations, the CCSD(T) [71,72,73,74,75], using all basis sets from the theoretical analysis given in the previous section, including pecG-*n* (*n* = 1, 2). Then, the vibrational and relativistic corrections were added to the basic values. The vibrational corrections were evaluated within the CCSD method [76,77,78,79,80] as the differences: Δ*r_vib_* = *r*_eff_ − *r*_e_, (where *r*_eff_ is either *r*_g_ or *r*_a_), using the first-level basis sets of the corresponding type. The relativistic corrections Δ*r_rel_* were evaluated at the 4-component DFT(PBE0 [81,82,83]) level as the differences between the relativistic and approximated nonrelativistic values: Δ*r_rel_* = *r*_rel_ − *r*_nrel_. For the estimation of Δ*r_rel_*, the dyall.v3z basis set [84,85] was applied. For more information about the calculations of relativistic corrections, please, see the “Materials and Methods” section. All calculated equilibrium bond lengths of molecules **1**–**18**, their vibrational and relativistic corrections, and corresponding experimental values are given in Tables S5–S12.

**Figure 2:**
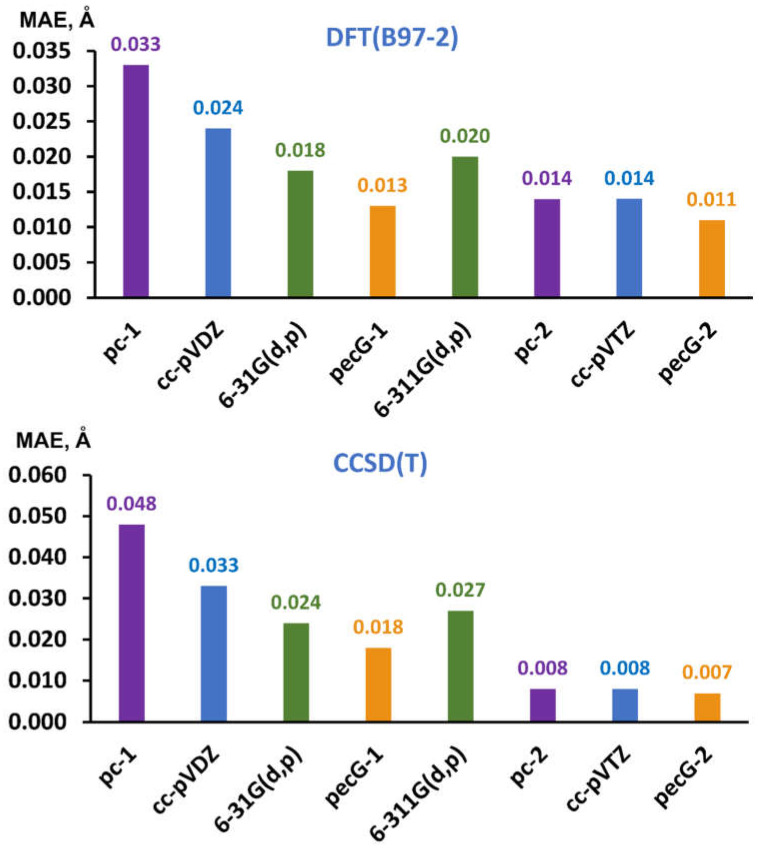
MAEs (in Å) for the equilibrium bond lengths in molecules **1**–**18** obtained in the optimization of their geometrical structures performed within the DFT(B97-2) (upper diagram) and CCSD(T) (bottom diagram) levels of theory using different basis sets, with taking into account relativistic and vibrational corrections, evaluated against corresponding experimental reference data.

The MAE for each considered basis set was calculated analogously to that in the theoretical analysis, but with the reference data were taken from the GED experiment. The calculated MAEs are shown in Figure 2.

Figure 2 vividly demonstrates a superior performance of our new pecG-*n* (*n* = 1, 2) basis sets over the usual energy-optimized basis sets for both cardinal numbers within all considered levels of electronic theory. The 6-31G(d,p) basis set again demonstrates the second-best performance among the first-level basis sets, surpassing the 6-311G(d,p) basis set in the accuracy of the results for both the DFT(B97-2) and CCSD(T) methods. It is interesting to note that the values calculated within the CCSD(T)/pecG-2 scheme are characterized by the MAE of only 0.007 Å, which is very close to the average range of uncertainty of the experimental values, being 0.005 Å for the data under consideration.

**Figure 3:**
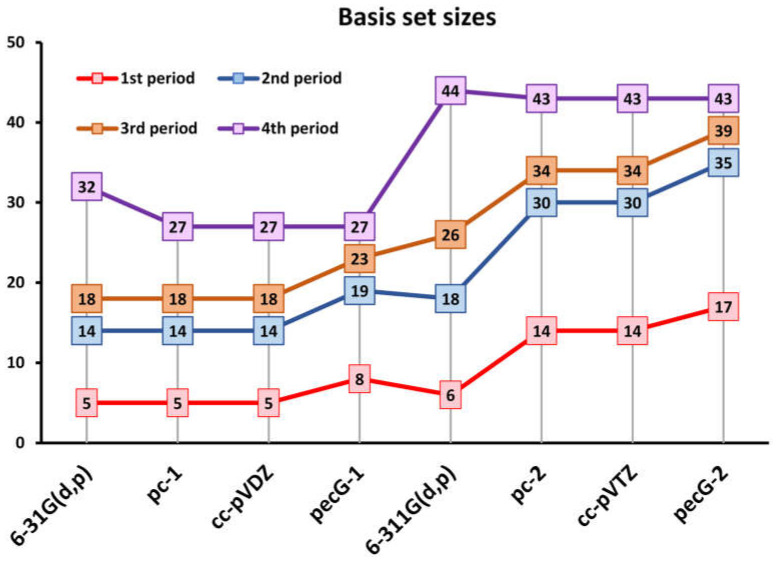
Number of basis set functions for the considered basis sets for 1–4 period atoms.

Thus, in all tests, new pecG-*n* (*n* = 1, 2) basis sets demonstrated a superior accuracy over all considered standard energy-optimized basis sets. Moreover, it is worth mentioning that newly optimized pecG-*n* basis sets for the 4th period atoms are of the same sizes as the pc-*n* and cc-pVXZ basis sets for both double- and triple-zeta levels, numbering 27 and 43 basis functions, respectively. At the same time, the 6-31G(d,p) basis set is even five functions larger than the pecG-1, pc-1 and cc-pVDZ basis sets, and the 6-311G(d,p) is one function larger than the pecG-2, pc-2 and cc-pVTZ basis sets. For the sake of convenience, we have plotted the number of basis set functions for each considered basis set for atoms of 1–4 periods in Figure 3, so that one could estimate the size of the basis functional space for an arbitrary molecule.

### 3.3. Testing the Performance of Equilibrium Geometries Obtained with the pecG-n (n = 1, 2) Basis Sets on Polarizability

Our previous tests were mostly concentrated on the equilibrium bond lengths, their results speak in favor of the pecG-*n* (*n* = 1, 2) basis sets as being the most accurate and efficient basis sets for these parameters, however, the quality of the whole equilibrium geometry provided by new basis sets can be unambiguously probed by the calculation of a molecular property that is drastically sensitive to the whole lot of geometry parameters of a molecule, including bond lengths, valence bond angles, and dihedral angles. Thus, we have chosen the static dipole polarizability to represent the property that defies our basis sets, because this property is known to be very sensitive to the whole geometry on which it is calculated [86,87,88,89]. Indeed, the problem of accurate prediction of linear optical properties is still sound, and static dipole polarizability is usually considered as a natural prerequisite property for elaborating computational methodologies for more complex static and dynamic optical properties. Static dipole polarizability is a second-order linear response electric property that is calculated through the sum-over-states expression over the singlet excited states of a molecule, which are essentially sensitive to all geometric parameters.

We have calculated static dipole polarizabilities of molecules **1**−**18** at the DFT(B97-2)/aug-cc-pVQZ level of theory on different equilibrium geometries obtained within the DFT(B97-2) method with various basis sets, including newly developed pecG-*n* (*n* = 1, 2) basis sets used on Ga−Br (and previously developed pecG-*n* (*n* = 1, 2) basis sets used for the rest atoms). In choosing the basis set for the calculation of dipole polarizabilities, we have relied upon the findings presented in the recent paper of Sauer et al. [90]. The reference theoretical data are calculated on the equilibrium geometries obtained with the cc-pV5Z basis set. All calculated polarizabilities are given in the Supplementary Materials in Table S13.

**Figure 4:**
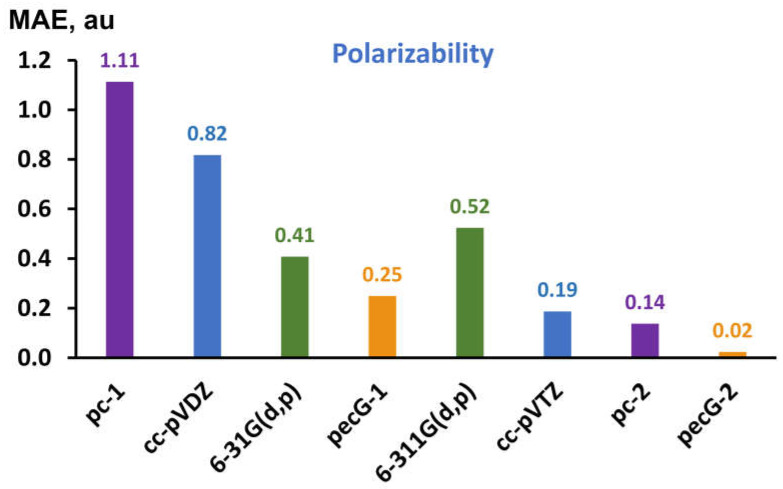
The MAEs (in au) for static dipole polarizabilities of molecules **1**–**18** that were calculated at the DFT(B97-2)/aug-cc-pVQZ level of theory on the equilibrium geometries obtained with different basis sets, evaluated against the corresponding theoretical reference data.

For polarizability values, we have calculated the MAEs against the theoretical reference data. The calculated MAEs are presented in Figure 4.

As can be seen from Figure 4, the least geometry factor error is provided by the pecG-1 basis set among the considered first-level basis sets, and by the pecG-2 basis set among the second-level basis sets. This unequivocally signifies that the equilibrium geometries obtained with our new pecG-*n* basis sets give the best values of polarizability calculated upon them, with the pecG-2 basis set providing the geometry factor error almost indistinguishable from that provided by the cc-pV5Z basis set (the MAE for polarizability is only 0.02). At the same time, Figure 4 testifies that the first-level pecG-1 basis set is not only the best one among the commensurate basis sets of double-zeta quality, but also surpasses the 6-311G(d,p) basis set of triple-zeta quality and gives the geometry factor error comparable to that provided by the triple-zeta cc-pVTZ basis set. In all this, it is pertinent to bear in mind that the pecG-*n* (*n* = 1, 2) basis sets for 4th period atoms are of the same sizes with the pc-*n* and cc-pVXZ basis sets of both doble- and triple-zeta levels, respectively, and the 6-31G(d,p) and 6-311G(d,p) basis sets are even larger.

## 4. Materials and Methods

The optimization of structures of molecules **1**–**18** was carried out within the DFT and MP2 methods using various basis sets in gas phase within the Gaussian 09 program (version C.01) [91]. The CCSD(T) and vibrationally averaged CCSD geometries for compounds **1**–**18** were obtained in the gas phase using the CFOUR program (version v2.1) [92]. Relativistic 4-component DFT geometry optimizations of molecules **1**–**18** were performed within the DIRAC code (release 14) [93]. The relativistic 4-component calculation of equilibrium geometry parameters was carried out using the full Dirac-Kohn-Sham Hamiltonian intrinsically covering both scalar and spin-orbit relativistic effects. In order to estimate nonrelativistic values of equilibrium bond lengths without leaving the 4-component representation, nonrelativistic 4-component Levy-Leblond approximation scheme [94] has been used. In all 4-component calculations, restricted kinetic balance (RKB) [95] has been applied. The static dipole polarizabilities were calculated using the Gaussian 09 program (version C.01). All calculations were carried out in A. E. Favorsky Irkutsk Institute of Chemistry on the facilities of the Baikal Analytical Centre (https://ckp-rf.ru/catalog/ckp/3050/, accessed on 20 July 2025).

## 5. Conclusions

In this paper, we have proposed new geometry-oriented pecG-*n* (*n* = 1, 2) basis sets for 4th-period p-elements, namely, Ga, Ge, As, Se, and Br. These basis sets were optimized via the property-energy consistent (PEC) algorithm directed to the minimization of the molecular energy gradient relative to the bond lengths. The presented basis sets demonstrated an outstanding performance in the geometry optimization of various molecules including Ga, Ge, As, Se, and Br elements as compared to the other popular commensurate basis sets of double- and triple-zeta quality that are frequently used for the calculation of equilibrium geometries.

The tests for new basis sets have been performed based not only on comparison of calculated equilibrium bond lengths with theoretical reference data, but also on the comparison with the values obtained in the gas phase electron diffraction experiment. The quality of equilibrium geometries obtained with the newly presented basis sets has also been tested against the other popular basis sets on the example of static dipole polarizability. The latter test, in particular, has unambiguously demonstrated that newly proposed pecG-*n* (*n* = 1, 2) basis sets provide the least geometry factor error relative to the data obtained with the reference geometry of the cc-pV5Z level. Even more so, the pecG-2 basis set used at the geometry optimization stage has provided the MAE for static dipole polarizability of only 0.02 au. This is a very encouraging result, as it speaks of the pecG-2 as the basis set that provides the equilibrium geometries of so high quality that the difference with the cc-pV5Z basis set is practically nought.

## Data Availability

The original contributions presented in this study are included in the article/Supplementary Materials. Further inquiries can be directed to the corresponding authors.

## Supplementary Materials

The following supporting information can be downloaded at: https://www.mdpi.com/article/10.3390/ijms26178197/s1.
