# Supplementary material for: Efficient pecG-n (n = 1, 2) Basis Sets for Ga, Ge, As, Se, and Br Specialized for the Geometry Optimization of Molecular Structures
Source: Int J Mol Sci. 2025 Aug 23;26(17):8197. doi: 10.3390/ijms26178197 (PMC12428671; doi:10.3390/ijms26178197)
Supplement: Supplementary file 1 [file ijms-26-08197-s001.zip › ijms-3821959-supplementary.pdf]

Supplementary Information

Efficient pecG-*n* (*n* = 1, 2) Basis Sets for Ga, Ge, As, Se, and Br specialized for the Geometry Optimization of Molecular Structures

By *Yuriy Yu. Rusakov* and *Irina L. Rusakova*

Table of contents

pecG-1 for Ga, Ge, As, Se, Br (Gaussian Format) 2
pecG-2 for Ga, Ge, As, Se, Br (Gaussian Format) 16

Table S1. Equilibrium bond lengths (in Å) in molecules **1-18** calculated at the MP2 level of theory with different basis sets.. 34

Table S2. Equilibrium bond lengths (in Å) in molecules **1-18** calculated at the DFT(B97-2) level of theory with different basis sets 35

Table S3. Equilibrium valence bond angles (in arc degrees, °) in molecules **1-18** calculated at the MP2 level of theory with different basis sets taking into account vibrational and relativistic corrections 36

Table S4. Equilibrium valence bond angles (in arc degrees, °) in molecules **1-18** calculated at the DFT(B97-2) level of theory with different basis sets taking into account vibrational and relativistic corrections. 36

Table S5. Total bond lengths (in Å) in molecules **1-18** calculated at the CCSD(T) level of theory with the pecG-1 and pecG-2 basis sets taking into account vibrational and relativistic corrections, against experiment... 37

Table S6. Total bond lengths (in Å) in molecules **1-18** calculated at the CCSD(T) level of theory with the cc-pVDZ and cc-pVTZ basis sets taking into account vibrational and relativistic corrections, against experiment 37

Table S7. Total bond lengths (in Å) in molecules **1-18** calculated at the CCSD(T) level of theory with the 6-31G(d,p) and 6-311G(d,p) basis sets taking into account vibrational and relativistic corrections, against experiment 38

Table S8. Total bond lengths (in Å) in molecules **1-18** calculated at the CCSD(T) level of theory with the pc-1 and pc-2 basis sets taking into account vibrational and relativistic corrections, against experiment.. 39

Table S9. Total bond lengths (in Å) in molecules **1-18** calculated at the DFT(B97-2) level of theory with the pecG-1 and pecG-2 basis sets taking into account vibrational and relativistic corrections, against experiment.. 40

Table S10. Total bond lengths (in Å) in molecules **1-18** calculated at the DFT(B97-2) level of theory with the cc-pVDZ and cc-pVTZ basis sets taking into account vibrational and relativistic corrections, against experiment 41

Table S11. Total bond lengths (in Å) in molecules **1-18** calculated at the DFT(B97-2) level of theory with the 6-31G(d,p) and 6-311G(d,p) basis sets taking into account vibrational and relativistic corrections, against experiment...................... 41

Table S12. Total bond lengths (in Å) in molecules **1-18** calculated at the DFT(B97-2) level of theory with the pc-1 and pc-2 basis sets taking into account vibrational and relativistic corrections, against experiment.. 42

Table S13. Static dipole polarizability (in au) calculated at the DFT(B97-2)/aug-cc-pVQZ level on different equilibrium geometries obtained within the DFT(B97-2) method with various basis sets, including the pecG-*n* (*n* = 1, 2)........................... 43

pecG-1 for Ga, Ge, As, Se, Br (Gaussian Format)

```
Ga     0
S   13   1.00
      4.876542E+05         2.010433E-04
      7.413831E+04         1.574689E-03
      1.634350E+04         8.650487E-03
      4.543491E+03         3.516298E-02
      1.500578E+03         1.111674E-01
      5.476257E+02         2.735210E-01
      2.142159E+02         4.188830E-01
      8.755391E+01         2.715168E-01
      2.780853E+01         2.694438E-02
      1.168454E+01        -5.141730E-03
```

3.292092E+00	1.686178E-03
1.434291E+00	-8.267898E-04
2.013480E-01	1.665020E-04

S 13 1.00

4.876542E+05	-6.090815E-05
7.413831E+04	-4.861692E-04
1.634350E+04	-2.708727E-03
4.543491E+03	-1.095631E-02
1.500578E+03	-3.730643E-02
5.476257E+02	-1.005953E-01
2.142159E+02	-2.118013E-01
8.755391E+01	-1.760523E-01
2.780853E+01	4.586901E-01
1.168454E+01	6.626861E-01
3.292092E+00	7.364541E-02
1.434291E+00	-1.423500E-02
2.013480E-01	2.698965E-03

S 13 1.00

4.876542E+05	2.013683E-05
7.413831E+04	1.901291E-04
1.634350E+04	1.016903E-03
4.543491E+03	4.276451E-03
1.500578E+03	1.404622E-02
5.476257E+02	4.001652E-02
2.142159E+02	8.412050E-02
8.755391E+01	8.168427E-02
2.780853E+01	-2.874315E-01
1.168454E+01	-5.270266E-01
3.292092E+00	5.325940E-01
1.434291E+00	6.929712E-01
2.013480E-01	4.679129E-02

S 13 1.00

4.876542E+05	-5.104743E-06
7.413831E+04	-4.105207E-05
1.634350E+04	-1.998690E-04
4.543491E+03	-8.214348E-04
1.500578E+03	-2.772700E-03
5.476257E+02	-7.701803E-03

2.142159E+02	-1.638920E-02
8.755391E+01	-1.685791E-02
2.780853E+01	6.262376E-02
1.168454E+01	1.075889E-01
3.292092E+00	-1.244016E-01
1.434291E+00	-2.994271E-01
2.013480E-01	4.686600E-01

S 1 1.00

7.072855E-02	1.000000E+00

P 10 1.00

3.291870E+03	1.480272E-03
7.713229E+02	1.268582E-02
2.522347E+02	6.089136E-02
9.547688E+01	2.001312E-01
3.892778E+01	4.068157E-01
1.649877E+01	3.934258E-01
6.817222E+00	1.060648E-01
2.830681E+00	2.772046E-04
1.100045E+00	1.821849E-03
2.503498E-01	-3.171576E-04

P 10 1.00

3.291870E+03	-5.726672E-04
7.713229E+02	-4.824373E-03
2.522347E+02	-2.383722E-02
9.547688E+01	-8.209164E-02
3.892778E+01	-1.797949E-01
1.649877E+01	-1.582261E-01
6.817222E+00	2.419174E-01
2.830681E+00	5.927571E-01
1.100045E+00	3.198415E-01
2.503498E-01	1.617940E-02

P 10 1.00

3.291870E+03	7.866828E-05
7.713229E+02	6.720020E-04
2.522347E+02	3.458974E-03
9.547688E+01	1.159928E-02
3.892778E+01	2.631556E-02
1.649877E+01	2.213860E-02

```
     6.817222E+00    -4.114163E-02
     2.830681E+00    -1.081710E-01
     1.100045E+00    -5.308376E-02
     2.503498E-01     3.388572E-01
P   1   1.00
     6.480297E-02     1.000000E+00
D   5   1.00
     6.729650E+01     2.551041E-02
     1.939441E+01     1.396915E-01
     6.718145E+00     3.474285E-01
     2.368335E+00     4.504233E-01
     7.569875E-01     3.304716E-01
D   1   1.00
     2.025899E-01     1.000000E+00
****

Ge     0
S   13   1.00
     5.331753E+05     1.993397E-04
     7.815634E+04     1.604489E-03
     1.780496E+04     8.240293E-03
     5.006087E+03     3.417736E-02
     1.627625E+03     1.116250E-01
     5.875365E+02     2.744369E-01
     2.300924E+02     4.175293E-01
     9.487472E+01     2.766709E-01
     2.918290E+01     2.967030E-02
     1.238090E+01    -7.242519E-03
     3.772911E+00     2.580780E-03
     1.578757E+00    -1.188427E-03
     2.713388E-01     2.797356E-04
S   13   1.00
     5.331753E+05    -5.995039E-05
     7.815634E+04    -4.896532E-04
     1.780496E+04    -2.620150E-03
     5.006087E+03    -1.071039E-02
     1.627625E+03    -3.798419E-02
     5.875365E+02    -1.009292E-01
```

```
     2.300924E+02        -2.130953E-01
     9.487472E+01        -1.751311E-01
     2.918290E+01         4.676784E-01
     1.238090E+01         6.332684E-01
     3.772911E+00         7.096758E-02
     1.578757E+00        -1.069587E-02
     2.713388E-01         2.170390E-03
S   13   1.00
     5.331753E+05         1.992639E-05
     7.815634E+04         1.902337E-04
     1.780496E+04         1.000266E-03
     5.006087E+03         4.187778E-03
     1.627625E+03         1.473009E-02
     5.875365E+02         4.059944E-02
     2.300924E+02         8.614189E-02
     9.487472E+01         8.143597E-02
     2.918290E+01        -2.976620E-01
     1.238090E+01        -5.340537E-01
     3.772911E+00         5.251300E-01
     1.578757E+00         7.427210E-01
     2.713388E-01         4.232776E-02
S   13   1.00
     5.331753E+05        -9.993355E-06
     7.815634E+04        -3.946840E-05
     1.780496E+04        -2.119759E-04
     5.006087E+03        -8.995294E-04
     1.627625E+03        -3.140193E-03
     5.875365E+02        -8.757172E-03
     2.300924E+02        -1.799956E-02
     9.487472E+01        -1.868659E-02
     2.918290E+01         7.181472E-02
     1.238090E+01         1.185604E-01
     3.772911E+00        -1.322961E-01
     1.578757E+00        -3.648879E-01
     2.713388E-01         5.134144E-01
S    1   1.00
     8.680092E-02         1.000000E+00
P   10   1.00
```

3.528157E+03	1.450346E-03
8.485540E+02	1.201295E-02
2.722635E+02	6.120424E-02
1.015378E+02	2.012317E-01
4.176665E+01	3.975457E-01
1.793812E+01	3.900599E-01
7.479848E+00	1.076506E-01
3.117093E+00	3.059155E-04
1.233887E+00	1.909194E-03
3.117157E-01	-3.908217E-04

P 10 1.00

3.528157E+03	-5.683090E-04
8.485540E+02	-4.810982E-03
2.722635E+02	-2.486442E-02
1.015378E+02	-8.540658E-02
4.176665E+01	-1.824489E-01
1.793812E+01	-1.626383E-01
7.479848E+00	2.331858E-01
3.117093E+00	5.832144E-01
1.233887E+00	3.296825E-01
3.117157E-01	1.654510E-02

P 10 1.00

3.528157E+03	9.045569E-05
8.485540E+02	7.095047E-04
2.722635E+02	3.807688E-03
1.015378E+02	1.280274E-02
4.176665E+01	2.826469E-02
1.793812E+01	2.399538E-02
7.479848E+00	-4.258905E-02
3.117093E+00	-1.136117E-01
1.233887E+00	-5.228026E-02
3.117157E-01	3.112645E-01

P 1 1.00

9.578430E-02	1.000000E+00

D 5 1.00

7.570884E+01	2.515374E-02
2.194158E+01	1.385600E-01
7.742316E+00	3.493967E-01

2.795105E+00 4.518937E-01
9.367726E-01 3.156406E-01
D 1 1.00
2.610972E-01 1.000000E+00
****

As 0
S 13 1.00
5.784430E+05 1.998119E-04
8.388458E+04 1.592160E-03
1.911642E+04 8.054648E-03
5.493521E+03 3.273720E-02
1.776242E+03 1.093319E-01
6.378209E+02 2.718683E-01
2.495846E+02 4.141873E-01
1.033654E+02 2.842586E-01
3.208360E+01 3.371582E-02
1.356462E+01 -7.986539E-03
3.960940E+00 2.892381E-03
1.630730E+00 -1.352969E-03
2.886295E-01 3.765994E-04
S 13 1.00
5.784430E+05 -5.936638E-05
8.388458E+04 -4.929992E-04
1.911642E+04 -2.572543E-03
5.493521E+03 -1.046240E-02
1.776242E+03 -3.745871E-02
6.378209E+02 -1.010559E-01
2.495846E+02 -2.123419E-01
1.033654E+02 -1.848566E-01
3.208360E+01 4.496059E-01
1.356462E+01 6.654582E-01
3.960940E+00 7.500071E-02
1.630730E+00 -1.321755E-02
2.886295E-01 3.149125E-03
S 13 1.00
5.784430E+05 1.997591E-05
8.388458E+04 1.906820E-04

```
     1.911642E+04        9.809635E-04
     5.493521E+03        4.021605E-03
     1.776242E+03        1.433495E-02
     6.378209E+02        3.974532E-02
     2.495846E+02        8.590433E-02
     1.033654E+02        8.473645E-02
     3.208360E+01       -2.843944E-01
     1.356462E+01       -5.626179E-01
     3.960940E+00        5.732406E-01
     1.630730E+00        6.994218E-01
     2.886295E-01        2.675321E-02
S   13   1.00
     5.784430E+05       -1.013885E-05
     8.388458E+04       -4.021513E-05
     1.911642E+04       -2.277093E-04
     5.493521E+03       -9.248054E-04
     1.776242E+03       -3.299903E-03
     6.378209E+02       -9.428214E-03
     2.495846E+02       -1.994944E-02
     1.033654E+02       -2.015184E-02
     3.208360E+01        6.871593E-02
     1.356462E+01        1.457751E-01
     3.960940E+00       -1.819493E-01
     1.630730E+00       -3.656127E-01
     2.886295E-01        5.998550E-01
S    1   1.00
     1.098827E-01        1.000000E+00
P   10   1.00
     3.850357E+03        1.429203E-03
     9.038234E+02        1.237733E-02
     2.889416E+02        6.148663E-02
     1.095093E+02        1.973637E-01
     4.545985E+01        3.955603E-01
     1.966607E+01        3.916492E-01
     8.377094E+00        1.153852E-01
     3.510791E+00        1.937109E-03
     1.476178E+00        1.664969E-03
     3.448975E-01       -2.614300E-04
```

```
P   10   1.00
     3.850357E+03          -5.637266E-04
     9.038234E+02          -4.963074E-03
     2.889416E+02          -2.533352E-02
     1.095093E+02          -8.515173E-02
     4.545985E+01          -1.814839E-01
     1.966607E+01          -1.724451E-01
     8.377094E+00           2.252441E-01
     3.510791E+00           5.746779E-01
     1.476178E+00           3.513750E-01
     3.448975E-01          2.667531E-02
P   10   1.00
     3.850357E+03           9.078602E-05
     9.038234E+02           7.838225E-04
     2.889416E+02           4.125737E-03
     1.095093E+02           1.359694E-02
     4.545985E+01           3.025382E-02
     1.966607E+01           2.643590E-02
     8.377094E+00          -4.068581E-02
     3.510791E+00          -1.266595E-01
     1.476178E+00          -5.132657E-02
     3.448975E-01          3.588187E-01
P    1   1.00
     1.083095E-01          1.000000E+00
D    5   1.00
     8.600670E+01           2.365515E-02
     2.487137E+01           1.362651E-01
     8.688504E+00           3.535399E-01
     3.111683E+00           4.757995E-01
     9.829019E-01          3.000412E-01
D    1   1.00
     3.279028E-01          1.000000E+00
****

Se     0
S   13   1.00
     6.006966E+05           2.024296E-04
     8.860337E+04           1.572450E-03
```

2.018514E+04	8.259905E-03
5.711171E+03	3.342064E-02
1.861182E+03	1.096733E-01
6.801288E+02	2.646726E-01
2.698700E+02	4.111545E-01
1.106406E+02	2.925183E-01
3.284882E+01	3.305120E-02
1.407395E+01	-8.691320E-03
4.447480E+00	3.300019E-03
1.830795E+00	-1.451767E-03
3.242753E-01	4.164359E-04

S 13 1.00

6.006966E+05	-6.047835E-05
8.860337E+04	-4.886751E-04
2.018514E+04	-2.575456E-03
5.711171E+03	-1.069742E-02
1.861182E+03	-3.748496E-02
6.801288E+02	-9.716614E-02
2.698700E+02	-2.126543E-01
1.106406E+02	-1.824519E-01
3.284882E+01	4.854831E-01
1.407395E+01	6.370183E-01
4.447480E+00	7.036797E-02
1.830795E+00	-9.250712E-03
3.242753E-01	2.096522E-03

S 13 1.00

6.006966E+05	2.010179E-05
8.860337E+04	1.879692E-04
2.018514E+04	1.054728E-03
5.711171E+03	4.181874E-03
1.861182E+03	1.477046E-02
6.801288E+02	3.959980E-02
2.698700E+02	8.763474E-02
1.106406E+02	8.450624E-02
3.284882E+01	-3.105772E-01
1.407395E+01	-5.574864E-01
4.447480E+00	5.561670E-01
1.830795E+00	7.550112E-01

3.242753E-01 3.175769E-02
S 13 1.00
6.006966E+05 -9.787188E-06
8.860337E+04 -6.065034E-05
2.018514E+04 -2.982616E-04
5.711171E+03 -1.235247E-03
1.861182E+03 -4.269103E-03
6.801288E+02 -1.159926E-02
2.698700E+02 -2.596341E-02
1.106406E+02 -2.577253E-02
3.284882E+01 9.372103E-02
1.407395E+01 1.828420E-01
4.447480E+00 -2.344297E-01
1.830795E+00 -4.599785E-01
3.242753E-01 7.564504E-01
S 1 1.00
1.252397E-01 1.000000E+00
P 10 1.00
4.128503E+03 1.450445E-03
9.643927E+02 1.207674E-02
3.154182E+02 5.832317E-02
1.202964E+02 1.907741E-01
4.994588E+01 3.893496E-01
2.163374E+01 3.984914E-01
9.085810E+00 1.238259E-01
3.887536E+00 6.468025E-04
1.612111E+00 2.380905E-03
4.181497E-01 -5.099128E-04
P 10 1.00
4.128503E+03 -5.751987E-04
9.643927E+02 -4.688751E-03
3.154182E+02 -2.410072E-02
1.202964E+02 -8.098949E-02
4.994588E+01 -1.781332E-01
2.163374E+01 -1.723545E-01
9.085810E+00 2.088493E-01
3.887536E+00 5.762119E-01
1.612111E+00 3.500040E-01

```
     4.181497E-01          2.320935E-02
P   10   1.00
     4.128503E+03          1.379459E-04
     9.643927E+02          1.128323E-03
     3.154182E+02          5.907051E-03
     1.202964E+02          2.021760E-02
     4.994588E+01          4.454826E-02
     2.163374E+01          4.020805E-02
     9.085810E+00         -5.994505E-02
     3.887536E+00         -2.197648E-01
     1.612111E+00         -6.275894E-02
     4.181497E-01          4.724958E-01
P    1   1.00
     1.503567E-01          1.000000E+00
D    5   1.00
     9.820838E+01          2.165785E-02
     2.882274E+01          1.240490E-01
     1.032037E+01          3.369501E-01
     3.847675E+00          4.526440E-01
     1.394899E+00          3.313206E-01
D    1   1.00
     3.973521E-01          1.000000E+00
****

Br     0
S   13   1.00
     5.961970E+05          2.100227E-04
     9.517389E+04          1.504944E-03
     2.225147E+04          7.848289E-03
     6.246249E+03          3.238404E-02
     2.043444E+03          1.058693E-01
     7.366525E+02          2.668789E-01
     2.860910E+02          4.207563E-01
     1.163468E+02          2.893709E-01
     3.651297E+01          3.072715E-02
     1.540605E+01         -6.755207E-03
     4.800674E+00          2.410680E-03
     2.005060E+00         -1.092311E-03
```

3.612029E-01 2.786587E-04
S 13 1.00
5.961970E+05 -7.001597E-05
9.517389E+04 -4.710211E-04
2.225147E+04 -2.498428E-03
6.246249E+03 -1.029912E-02
2.043444E+03 -3.577682E-02
7.366525E+02 -9.853865E-02
2.860910E+02 -2.113339E-01
1.163468E+02 -1.913080E-01
3.651297E+01 4.558596E-01
1.540605E+01 6.559808E-01
4.800674E+00 7.435826E-02
2.005060E+00 -1.011852E-02
3.612029E-01 2.121706E-03
S 13 1.00
5.961970E+05 3.033845E-05
9.517389E+04 1.932507E-04
2.225147E+04 9.989487E-04
6.246249E+03 4.104804E-03
2.043444E+03 1.419788E-02
7.366525E+02 4.051399E-02
2.860910E+02 8.923244E-02
1.163468E+02 8.882801E-02
3.651297E+01 -2.914943E-01
1.540605E+01 -5.823648E-01
4.800674E+00 5.415654E-01
2.005060E+00 7.391331E-01
3.612029E-01 2.854130E-02
S 13 1.00
5.961970E+05 -1.007553E-05
9.517389E+04 -4.969580E-05
2.225147E+04 -2.863169E-04
6.246249E+03 -1.219013E-03
2.043444E+03 -4.159238E-03
7.366525E+02 -1.193289E-02
2.860910E+02 -2.639805E-02
1.163468E+02 -2.605851E-02

```
      3.651297E+01       9.105066E-02
      1.540605E+01       1.997894E-01
      4.800674E+00      -2.482168E-01
      2.005060E+00      -4.596050E-01
      3.612029E-01       7.696183E-01
S   1   1.00
      1.273901E-01       1.000000E+00
P  10   1.00
      4.300217E+03       1.437188E-03
      1.063306E+03       1.147475E-02
      3.439300E+02       5.795109E-02
      1.287503E+02       1.960266E-01
      5.266732E+01       4.001743E-01
      2.254609E+01       4.051655E-01
      9.208508E+00       1.133696E-01
      3.975958E+00      -3.192099E-03
      1.665050E+00       3.091689E-03
      4.521747E-01      -8.450513E-04
P  10   1.00
      4.300217E+03      -5.585561E-04
      1.063306E+03      -4.626089E-03
      3.439300E+02      -2.413602E-02
      1.287503E+02      -8.388264E-02
      5.266732E+01      -1.868145E-01
      2.254609E+01      -1.711204E-01
      9.208508E+00       2.403935E-01
      3.975958E+00       5.740080E-01
      1.665050E+00       3.115522E-01
      4.521747E-01       1.453682E-02
P  10   1.00
      4.300217E+03       1.459501E-04
      1.063306E+03       1.174300E-03
      3.439300E+02       6.340150E-03
      1.287503E+02       2.134451E-02
      5.266732E+01       5.136412E-02
      2.254609E+01       3.948108E-02
      9.208508E+00      -6.566441E-02
      3.975958E+00      -2.219541E-01
```

```
     1.665050E+00         -2.149853E-02
     4.521747E-01          5.158741E-01
P    1   1.00
     1.697658E-01          1.000000E+00
D    5   1.00
     1.201655E+02          1.787304E-02
     3.550523E+01          1.115030E-01
     1.283278E+01          3.149246E-01
     4.844736E+00          5.062184E-01
     1.764342E+00          3.219972E-01
D    1   1.00
     4.533873E-01          1.000000E+00
****
```

pecG-2 for Ga, Ge, As, Se, Br (Gaussian Format)

```
Ga     0
S   18   1.00
     6.455191E+06          9.996085E-06
     1.018555E+06          6.098190E-05
     2.252633E+05          3.151965E-04
     6.381974E+04          1.382626E-03
     2.055208E+04          4.887423E-03
     7.587891E+03          1.547112E-02
     2.955244E+03          4.522438E-02
     1.221034E+03          1.147424E-01
     5.224467E+02          2.416269E-01
     2.380786E+02          3.374990E-01
     1.138352E+02          2.943790E-01
     5.124703E+01          9.317600E-02
     2.457943E+01         -5.084380E-03
     1.163585E+01          4.760959E-03
     5.163932E+00         -2.669942E-03
     2.519200E+00          1.423380E-03
     1.120392E+00         -5.220698E-04
     1.252975E-01         -1.513167E-04
S   18   1.00
     6.455191E+06         -3.042630E-06
```

1.018555E+06 -2.050093E-05
2.252633E+05 -1.002107E-04
6.381974E+04 -4.380232E-04
2.055208E+04 -1.528626E-03
7.587891E+03 -4.964851E-03
2.955244E+03 -1.421767E-02
1.221034E+03 -3.950911E-02
5.224467E+02 -9.150357E-02
2.380786E+02 -1.610655E-01
1.138352E+02 -1.997616E-01
5.124703E+01 -1.902027E-03
2.457943E+01 5.087061E-01
1.163585E+01 5.536766E-01
5.163932E+00 1.033078E-01
2.519200E+00 -2.011989E-03
1.120392E+00 2.364193E-03
1.252975E-01 3.424620E-04
S 18 1.00
6.455191E+06 9.684828E-07
1.018555E+06 1.023221E-05
2.252633E+05 3.957726E-05
6.381974E+04 1.602651E-04
2.055208E+04 5.807315E-04
7.587891E+03 1.856981E-03
2.955244E+03 5.514079E-03
1.221034E+03 1.491102E-02
5.224467E+02 3.593421E-02
2.380786E+02 6.499067E-02
1.138352E+02 8.301838E-02
5.124703E+01 3.043467E-03
2.457943E+01 -3.141271E-01
1.163585E+01 -4.808791E-01
5.163932E+00 9.939910E-02
2.519200E+00 7.087270E-01
1.120392E+00 4.350081E-01
1.252975E-01 -9.849958E-03
S 18 1.00
6.455191E+06 -1.006347E-07

```
     1.018555E+06     -3.062587E-06
     2.252633E+05     -9.813912E-06
     6.381974E+04     -3.031193E-05
     2.055208E+04     -1.098294E-04
     7.587891E+03     -3.532924E-04
     2.955244E+03     -1.063973E-03
     1.221034E+03     -2.859986E-03
     5.224467E+02     -6.720443E-03
     2.380786E+02     -1.215911E-02
     1.138352E+02     -1.654621E-02
     5.124703E+01      1.993337E-04
     2.457943E+01      5.985194E-02
     1.163585E+01      1.071950E-01
     5.163932E+00     -3.517215E-02
     2.519200E+00     -1.744157E-01
     1.120392E+00     -2.401378E-01
     1.252975E-01      4.403819E-01
S   1   1.00
     2.638022E-01      1.000000E+00
S   1   1.00
     5.409997E-02      1.000000E+00
P  11   1.00
     7.873622E+03      3.231655E-04
     1.903149E+03      2.758886E-03
     6.152594E+02      1.519581E-02
     2.375647E+02      5.732753E-02
     1.022050E+02      1.628326E-01
     4.574157E+01      3.381116E-01
     2.085508E+01      3.982015E-01
     9.645525E+00      1.875162E-01
     4.511709E+00      1.879021E-02
     1.979990E+00      2.315388E-03
     2.065231E-01      1.234246E-04
P  11   1.00
     7.873622E+03     -1.205292E-04
     1.903149E+03     -1.067284E-03
     6.152594E+02     -5.988324E-03
     2.375647E+02     -2.293572E-02
```

```
    1.022050E+02     -6.751625E-02
    4.574157E+01     -1.486571E-01
    2.085508E+01     -1.789740E-01
    9.645525E+00      3.330259E-02
    4.511709E+00      4.161207E-01
    1.979990E+00      4.987481E-01
    2.065231E-01      4.761954E-03
P  11  1.00
    7.873622E+03      1.986375E-05
    1.903149E+03      1.488218E-04
    6.152594E+02      8.314777E-04
    2.375647E+02      3.155829E-03
    1.022050E+02      9.217533E-03
    4.574157E+01      2.031594E-02
    2.085508E+01      2.658392E-02
    9.645525E+00     -9.030877E-03
    4.511709E+00     -6.646504E-02
    1.979990E+00     -1.010471E-01
    2.065231E-01      3.781876E-01
P   1  1.00
    8.258963E-01      1.000000E+00
P   1  1.00
    5.666267E-02      1.000000E+00
D   7  1.00
    2.441387E+02      2.018080E-03
    7.405430E+01      1.579643E-02
    2.802349E+01      7.083026E-02
    1.147404E+01      1.986449E-01
    4.987842E+00      3.142681E-01
    2.147699E+00      3.846341E-01
    8.152255E-01      2.815434E-01
D   1  1.00
    3.529147E-01      1.000000E+00
D   1  1.00
    1.173199E-01      1.000000E+00
F   1  1.00
    3.540069E-01      1.000000E+00
****
```

Ge 0

S 18 1.00

7.344619E+06	9.967806E-06
1.126189E+06	5.997098E-05
2.544892E+05	3.003283E-04
7.373914E+04	1.209176E-03
2.387742E+04	4.711229E-03
8.337021E+03	1.540722E-02
3.272657E+03	4.289794E-02
1.345181E+03	1.132577E-01
5.784122E+02	2.332452E-01
2.631682E+02	3.450402E-01
1.247734E+02	2.954301E-01
6.121796E+01	9.733747E-02
2.827929E+01	7.170131E-03
1.365496E+01	-4.016834E-04
6.306024E+00	2.690230E-04
2.852705E+00	-2.030862E-04
1.230843E+00	6.027676E-05
1.650462E-01	2.024259E-05

S 18 1.00

7.344619E+06	-1.012137E-06
1.126189E+06	-2.007260E-05
2.544892E+05	-8.975060E-05
7.373914E+04	-3.824098E-04
2.387742E+04	-1.488197E-03
8.337021E+03	-4.844470E-03
3.272657E+03	-1.383649E-02
1.345181E+03	-3.818565E-02
5.784122E+02	-8.823794E-02
2.631682E+02	-1.600983E-01
1.247734E+02	-1.980534E-01
6.121796E+01	-2.389153E-02
2.827929E+01	4.356178E-01
1.365496E+01	5.737030E-01
6.306024E+00	1.450858E-01
2.852705E+00	1.338357E-03

1.230843E+00	2.329071E-03
1.650462E-01	4.531364E-04

S 18 1.00

7.344619E+06	5.031283E-07
1.126189E+06	9.929214E-06
2.544892E+05	3.987612E-05
7.373914E+04	1.411514E-04
2.387742E+04	5.598537E-04
8.337021E+03	1.898140E-03
3.272657E+03	5.269136E-03
1.345181E+03	1.439118E-02
5.784122E+02	3.427037E-02
2.631682E+02	6.383224E-02
1.247734E+02	8.426286E-02
6.121796E+01	1.138165E-02
2.827929E+01	-2.669739E-01
1.365496E+01	-5.046105E-01
6.306024E+00	6.937891E-03
2.852705E+00	7.606811E-01
1.230843E+00	4.565341E-01
1.650462E-01	-1.982911E-03

S 18 1.00

7.344619E+06	-3.006039E-07
1.126189E+06	-4.898531E-06
2.544892E+05	-9.900265E-06
7.373914E+04	-2.944539E-05
2.387742E+04	-1.194524E-04
8.337021E+03	-3.804241E-04
3.272657E+03	-1.141958E-03
1.345181E+03	-3.130176E-03
5.784122E+02	-7.222746E-03
2.631682E+02	-1.374384E-02
1.247734E+02	-1.794797E-02
6.121796E+01	-1.832339E-03
2.827929E+01	5.772837E-02
1.365496E+01	1.225424E-01
6.306024E+00	-1.138853E-02
2.852705E+00	-2.200124E-01

1.230843E+00	-2.824631E-01
1.650462E-01	4.857964E-01

S 1 1.00

3.432515E-01	1.000000E+00

S 1 1.00

7.181554E-02	1.000000E+00

P 11 1.00

7.043991E+03	4.487356E-04
1.645487E+03	3.985112E-03
5.368789E+02	2.110993E-02
2.055723E+02	7.930905E-02
8.629617E+01	2.183042E-01
3.826281E+01	3.852169E-01
1.742268E+01	3.589989E-01
7.864769E+00	1.043780E-01
3.463636E+00	4.681214E-03
1.478665E+00	1.420214E-03
2.121560E-01	1.337203E-04

P 11 1.00

7.043991E+03	-1.803029E-04
1.645487E+03	-1.539340E-03
5.368789E+02	-8.251889E-03
2.055723E+02	-3.211097E-02
8.629617E+01	-9.289800E-02
3.826281E+01	-1.753662E-01
1.742268E+01	-1.477583E-01
7.864769E+00	1.990706E-01
3.463636E+00	5.329087E-01
1.478665E+00	3.905724E-01
2.121560E-01	-3.762572E-03

P 11 1.00

7.043991E+03	2.933295E-05
1.645487E+03	2.254365E-04
5.368789E+02	1.207982E-03
2.055723E+02	4.789996E-03
8.629617E+01	1.359497E-02
3.826281E+01	2.645872E-02
1.742268E+01	2.299561E-02

```
    7.864769E+00        -3.522510E-02
    3.463636E+00        -1.042645E-01
    1.478665E+00        -7.916423E-02
    2.121560E-01         3.683412E-01
P   1   1.00
    5.420645E-01         1.000000E+00
P   1   1.00
    6.789575E-02         1.000000E+00
D   7   1.00
    2.705989E+02         1.864358E-03
    8.449328E+01         1.537982E-02
    3.173729E+01         6.902105E-02
    1.338106E+01         1.860687E-01
    5.959230E+00         3.179513E-01
    2.587524E+00         3.822734E-01
    1.037343E+00         2.641893E-01
D   1   1.00
    4.640567E-01         1.000000E+00
D   1   1.00
    1.534491E-01         1.000000E+00
F   1   1.00
    4.689773E-01         1.000000E+00
****

As   0
S  18   1.00
    8.599091E+06         9.623935E-06
    1.278396E+06         4.955926E-05
    2.887568E+05         2.747246E-04
    8.228112E+04         1.165778E-03
    2.632140E+04         4.203470E-03
    9.606867E+03         1.312831E-02
    3.815784E+03         3.732998E-02
    1.573799E+03         9.836073E-02
    6.795719E+02         2.094608E-01
    3.040024E+02         3.341463E-01
    1.436656E+02         2.951837E-01
    7.112581E+01         1.178051E-01
```

3.198936E+01	1.042673E-02
1.548402E+01	-5.337538E-04
7.164793E+00	2.437310E-04
3.380958E+00	-1.512041E-04
1.465562E+00	4.002820E-05
2.133420E-01	9.796705E-06

S 18 1.00

8.599091E+06	-3.017370E-06
1.278396E+06	-2.007122E-05
2.887568E+05	-8.883352E-05
8.228112E+04	-3.679133E-04
2.632140E+04	-1.350803E-03
9.606867E+03	-4.215285E-03
3.815784E+03	-1.271095E-02
1.573799E+03	-3.411871E-02
6.795719E+02	-8.162442E-02
3.040024E+02	-1.546009E-01
1.436656E+02	-2.011668E-01
7.112581E+01	-5.738042E-02
3.198936E+01	4.077550E-01
1.548402E+01	5.889912E-01
7.164793E+00	1.737388E-01
3.380958E+00	1.001187E-03
1.465562E+00	3.232584E-03
2.133420E-01	7.527529E-04

S 18 1.00

8.599091E+06	9.861504E-07
1.278396E+06	9.977361E-06
2.887568E+05	2.958659E-05
8.228112E+04	1.429395E-04
2.632140E+04	5.045835E-04
9.606867E+03	1.742867E-03
3.815784E+03	4.903305E-03
1.573799E+03	1.347392E-02
6.795719E+02	3.216054E-02
3.040024E+02	6.456269E-02
1.436656E+02	8.680719E-02
7.112581E+01	2.584564E-02

3.198936E+01	-2.491808E-01
1.548402E+01	-5.040268E-01
7.164793E+00	-7.904056E-02
3.380958E+00	6.918567E-01
1.465562E+00	5.351280E-01
2.133420E-01	-4.903712E-03

S 18 1.00

8.599091E+06	-1.038382E-07
1.278396E+06	-2.981539E-06
2.887568E+05	-9.432348E-06
8.228112E+04	-2.985445E-05
2.632140E+04	-1.264505E-04
9.606867E+03	-3.927243E-04
3.815784E+03	-1.066068E-03
1.573799E+03	-3.255096E-03
6.795719E+02	-7.222743E-03
3.040024E+02	-1.466709E-02
1.436656E+02	-1.829691E-02
7.112581E+01	-6.755460E-03
3.198936E+01	6.234791E-02
1.548402E+01	1.316342E-01
7.164793E+00	1.892950E-02
3.380958E+00	-2.336432E-01
1.465562E+00	-3.241984E-01
2.133420E-01	5.597298E-01

S 1 1.00

4.132273E-01	1.000000E+00

S 1 1.00

8.847790E-02	1.000000E+00

P 11 1.00

7.357957E+03	4.446750E-04
1.814394E+03	3.773027E-03
5.753943E+02	2.154822E-02
2.176341E+02	8.189087E-02
9.132630E+01	2.207421E-01
4.087704E+01	3.860462E-01
1.869948E+01	3.629274E-01
8.303275E+00	1.064059E-01

```
     3.747270E+00          2.412947E-03
     1.607993E+00          2.115868E-03
     2.332372E-01          3.173256E-04
P   11   1.00
     7.357957E+03         -1.708236E-04
     1.814394E+03         -1.532584E-03
     5.753943E+02         -8.378742E-03
     2.176341E+02         -3.343057E-02
     9.132630E+01         -9.389028E-02
     4.087704E+01         -1.759644E-01
     1.869948E+01         -1.452783E-01
     8.303275E+00          2.024989E-01
     3.747270E+00          5.325794E-01
     1.607993E+00          3.839417E-01
     2.332372E-01         -5.143658E-03
P   11   1.00
     7.357957E+03          2.953238E-05
     1.814394E+03          2.424010E-04
     5.753943E+02          1.326216E-03
     2.176341E+02          5.447533E-03
     9.132630E+01          1.468914E-02
     4.087704E+01          2.930619E-02
     1.869948E+01          2.338417E-02
     8.303275E+00         -3.870973E-02
     3.747270E+00         -1.096979E-01
     1.607993E+00         -8.291174E-02
     2.332372E-01          3.609322E-01
P    1   1.00
     5.502814E-01          1.000000E+00
P    1   1.00
     8.305407E-02          1.000000E+00
D    7   1.00
     3.230550E+02          1.652060E-03
     9.628806E+01          1.449978E-02
     3.590801E+01          6.689202E-02
     1.519280E+01          1.797137E-01
     6.876654E+00          3.125585E-01
     3.079870E+00          3.657375E-01
```

```
     1.334925E+00         2.702379E-01
D    1   1.00
     5.271344E-01         1.000000E+00
D    1   1.00
     1.917143E-01         1.000000E+00
F    1   1.00
     5.524175E-01         1.000000E+00
****

Se     0
S   18   1.00
     9.448960E+06         9.448260E-06
     1.496903E+06         5.045684E-05
     3.476325E+05         2.323834E-04
     9.834285E+04         1.030138E-03
     3.060100E+04         4.039939E-03
     1.070668E+04         1.310729E-02
     4.180892E+03         3.676534E-02
     1.759210E+03         9.175633E-02
     7.782356E+02         1.961823E-01
     3.545548E+02         3.261502E-01
     1.639286E+02         3.370969E-01
     7.541507E+01         1.331348E-01
     3.571288E+01         4.361514E-03
     1.702681E+01         3.109377E-03
     7.987420E+00        -1.699761E-03
     3.766938E+00         8.626882E-04
     1.698283E+00        -3.729005E-04
     2.499685E-01        -1.234208E-04
S   18   1.00
     9.448960E+06        -9.830186E-07
     1.496903E+06        -9.889833E-06
     3.476325E+05        -6.976905E-05
     9.834285E+04        -3.190771E-04
     3.060100E+04        -1.283965E-03
     1.070668E+04        -4.088270E-03
     4.180892E+03        -1.199243E-02
     1.759210E+03        -3.104187E-02
```

7.782356E+02	-7.295999E-02
3.545548E+02	-1.428628E-01
1.639286E+02	-2.107788E-01
7.541507E+01	-7.706905E-02
3.571288E+01	3.801571E-01
1.702681E+01	6.047719E-01
7.987420E+00	1.893161E-01
3.766938E+00	5.212493E-03
1.698283E+00	2.377513E-03
2.499685E-01	3.759954E-04

S 18 1.00

9.448960E+06	5.018017E-07
1.496903E+06	5.048743E-06
3.476325E+05	2.911110E-05
9.834285E+04	1.271763E-04
3.060100E+04	4.917575E-04
1.070668E+04	1.685443E-03
4.180892E+03	4.630968E-03
1.759210E+03	1.247733E-02
7.782356E+02	2.845819E-02
3.545548E+02	6.017535E-02
1.639286E+02	8.697773E-02
7.541507E+01	3.990478E-02
3.571288E+01	-2.308918E-01
1.702681E+01	-5.227893E-01
7.987420E+00	-9.704507E-02
3.766938E+00	7.002281E-01
1.698283E+00	5.390984E-01
2.499685E-01	-1.515157E-02

S 18 1.00

9.448960E+06	-1.984028E-07
1.496903E+06	-1.978515E-06
3.476325E+05	-9.974770E-06
9.834285E+04	-4.058888E-05
3.060100E+04	-1.515573E-04
1.070668E+04	-4.967650E-04
4.180892E+03	-1.346622E-03
1.759210E+03	-3.618705E-03

7.782356E+02	-8.383495E-03
3.545548E+02	-1.744369E-02
1.639286E+02	-2.667453E-02
7.541507E+01	-1.171947E-02
3.571288E+01	7.186907E-02
1.702681E+01	1.709422E-01
7.987420E+00	2.998423E-02
3.766938E+00	-2.842150E-01
1.698283E+00	-4.330722E-01
2.499685E-01	6.884474E-01

S 1 1.00

4.758211E-01	1.000000E+00

S 1 1.00

1.039036E-01	1.000000E+00

P 11 1.00

8.121111E+03	4.360651E-04
1.883053E+03	4.055568E-03
6.094586E+02	2.088939E-02
2.351662E+02	7.942657E-02
9.866374E+01	2.182539E-01
4.379095E+01	3.873095E-01
2.007037E+01	3.546551E-01
9.138895E+00	1.077155E-01
4.082280E+00	5.076356E-03
1.750560E+00	1.299814E-03
2.664132E-01	1.553494E-04

P 11 1.00

8.121111E+03	-1.836774E-04
1.883053E+03	-1.613185E-03
6.094586E+02	-8.676383E-03
2.351662E+02	-3.311210E-02
9.866374E+01	-9.642953E-02
4.379095E+01	-1.827585E-01
2.007037E+01	-1.489403E-01
9.138895E+00	1.902294E-01
4.082280E+00	5.414809E-01
1.750560E+00	3.716887E-01
2.664132E-01	-4.674575E-03

```
P   11   1.00
      8.121111E+03           3.905449E-05
      1.883053E+03           3.919940E-04
      6.094586E+02           2.043192E-03
      2.351662E+02           8.068557E-03
      9.866374E+01           2.269546E-02
      4.379095E+01           4.580840E-02
      2.007037E+01           3.267067E-02
      9.138895E+00          -5.562165E-02
      4.082280E+00          -1.805844E-01
      1.750560E+00          -1.009299E-01
      2.664132E-01           5.690571E-01
P    1   1.00
      5.887060E-01           1.000000E+00
P    1   1.00
      8.784063E-02           1.000000E+00
D    7   1.00
      3.469240E+02           1.728853E-03
      1.039437E+02           1.448938E-02
      3.989022E+01           6.429749E-02
      1.711302E+01           1.775984E-01
      7.778482E+00           3.123526E-01
      3.542755E+00           3.702279E-01
      1.539613E+00           2.754699E-01
D    1   1.00
      6.036912E-01           1.000000E+00
D    1   1.00
      2.286077E-01           1.000000E+00
F    1   1.00
      5.424075E-01           1.000000E+00
****

Br     0
S   18   1.00
      1.086292E+07           6.061740E-06
      1.605682E+06           3.997053E-05
      3.855579E+05           2.331222E-04
      1.036253E+05           1.031755E-03
```

3.318564E+04	3.781728E-03
1.189464E+04	1.202272E-02
4.790707E+03	3.299916E-02
1.968349E+03	9.184553E-02
8.253583E+02	2.086447E-01
3.693629E+02	3.222175E-01
1.770570E+02	3.184645E-01
8.622574E+01	1.441273E-01
3.944266E+01	1.179068E-02
1.939569E+01	1.067340E-03
1.012841E+01	-6.160299E-04
3.996478E+00	2.352859E-04
1.818844E+00	-1.620310E-04
3.086933E-01	-5.003983E-05

S 18 1.00

1.086292E+07	-1.975874E-06
1.605682E+06	-9.834981E-06
3.855579E+05	-7.049931E-05
1.036253E+05	-3.299295E-04
3.318564E+04	-1.200214E-03
1.189464E+04	-3.755186E-03
4.790707E+03	-1.068595E-02
1.968349E+03	-3.040187E-02
8.253583E+02	-7.739335E-02
3.693629E+02	-1.416519E-01
1.770570E+02	-1.952589E-01
8.622574E+01	-9.518278E-02
3.944266E+01	3.540756E-01
1.939569E+01	5.626090E-01
1.012841E+01	2.413393E-01
3.996478E+00	2.799691E-02
1.818844E+00	-5.359937E-03
3.086933E-01	-1.969003E-03

S 18 1.00

1.086292E+07	7.522309E-07
1.605682E+06	4.912800E-06
3.855579E+05	3.014456E-05
1.036253E+05	1.292352E-04

```
     3.318564E+04       4.730439E-04
     1.189464E+04       1.506474E-03
     4.790707E+03       4.356881E-03
     1.968349E+03       1.244941E-02
     8.253583E+02       3.140872E-02
     3.693629E+02       6.007208E-02
     1.770570E+02       8.701954E-02
     8.622574E+01       4.229549E-02
     3.944266E+01      -2.018348E-01
     1.939569E+01      -5.057488E-01
     1.012841E+01      -1.424592E-01
     3.996478E+00       7.101544E-01
     1.818844E+00       5.381859E-01
     3.086933E-01      -7.473447E-03
S   18   1.00
     1.086292E+07      -2.286530E-07
     1.605682E+06      -1.604951E-06
     3.855579E+05      -1.007509E-05
     1.036253E+05      -3.893187E-05
     3.318564E+04      -1.414532E-04
     1.189464E+04      -4.408400E-04
     4.790707E+03      -1.203188E-03
     1.968349E+03      -3.586480E-03
     8.253583E+02      -9.216690E-03
     3.693629E+02      -1.809930E-02
     1.770570E+02      -2.508871E-02
     8.622574E+01      -1.368675E-02
     3.944266E+01       6.559496E-02
     1.939569E+01       1.627696E-01
     1.012841E+01       5.534555E-02
     3.996478E+00      -3.239822E-01
     1.818844E+00      -3.819960E-01
     3.086933E-01       7.459416E-01
S    1   1.00
     5.720219E-01       1.000000E+00
S    1   1.00
     1.135524E-01       1.000000E+00
P   11   1.00
```

8.923021E+03	4.279690E-04
2.077500E+03	3.650727E-03
6.765727E+02	2.013836E-02
2.532265E+02	8.117938E-02
1.046765E+02	2.218943E-01
4.656848E+01	3.884915E-01
2.148817E+01	3.541697E-01
9.842682E+00	1.102504E-01
4.465203E+00	4.854118E-03
1.966672E+00	1.324043E-03
2.945077E-01	1.502396E-04

P 11 1.00

8.923021E+03	-1.667001E-04
2.077500E+03	-1.472831E-03
6.765727E+02	-8.308443E-03
2.532265E+02	-3.402599E-02
1.046765E+02	-9.791800E-02
4.656848E+01	-1.828384E-01
2.148817E+01	-1.493478E-01
9.842682E+00	1.969077E-01
4.465203E+00	5.434389E-01
1.966672E+00	3.846095E-01
2.945077E-01	-5.580948E-03

P 11 1.00

8.923021E+03	3.981741E-05
2.077500E+03	3.894000E-04
6.765727E+02	1.999495E-03
2.532265E+02	8.466062E-03
1.046765E+02	2.459880E-02
4.656848E+01	4.651826E-02
2.148817E+01	3.525556E-02
9.842682E+00	-6.264693E-02
4.465203E+00	-1.925981E-01
1.966672E+00	-1.153565E-01
2.945077E-01	5.572813E-01

P 1 1.00

6.814635E-01	1.000000E+00

P 1 1.00

1.024518E-01 1.000000E+00
D 7 1.00
3.963002E+02 1.587299E-03
1.158970E+02 1.377695E-02
4.512165E+01 5.900521E-02
1.976579E+01 1.656007E-01
8.995113E+00 3.114495E-01
4.029220E+00 3.846693E-01
1.732770E+00 2.700983E-01
D 1 1.00
7.114346E-01 1.000000E+00
D 1 1.00
2.788207E-01 1.000000E+00
F 1 1.00
6.009579E-01 1.000000E+00
****

Table S1. Equilibrium bond lengths (in Å) in molecules **1-18** calculated at the MP2 level of theory with different basis sets.

Molecule	Bond	cc-pV5Z	pecG-1	pecG-2	cc-pVDZ	cc-pVTZ	6-31G(d,p)	6-311G(d,p)	pc-1	pc-2
As_4 (**1**)	As-As	2.4043	2.4420	2.4100	2.4543	2.4166	2.4560	2.4596	2.4949	2.4190
$AsBr_3$ (**2**)	As-Br	2.2997	2.3204	2.3085	2.3446	2.3117	2.3462	2.3428	2.3776	2.3161
$AsCl_3$ (**3**)	As-Cl	2.1405	2.1525	2.1429	2.1808	2.1524	2.1753	2.1771	2.2053	2.1470
AsF_3 (**4**)	As-F	1.7015	1.7300	1.7047	1.7402	1.7035	1.7222	1.7343	1.7619	1.7032
$CHBr_3$ (**5**)	C-Br	1.9043	1.9266	1.9088	1.9364	1.9121	1.9366	1.9293	1.9486	1.9138
$GaBr_3$ (**6**)	Ga-Br	2.2226	2.2485	2.2266	2.2730	2.2315	2.2825	2.2580	2.2873	2.2370
$GaCl_3$ (**7**)	Ga-Cl	2.0835	2.1030	2.0854	2.1284	2.0956	2.1285	2.1066	2.1336	2.0910
$GeBr_2$ (**8**)	Ge-Br	2.3039	2.3179	2.3080	2.3462	2.3109	2.3527	2.3407	2.3769	2.3162
$O(GeH_3)_2$ (**9**)	Ge-O	1.7613	1.7837	1.7546	1.7953	1.7580	1.7970	1.7820	1.8087	1.7626
	Ge-H	1.5024	1.5352	1.5007	1.5340	1.5124	1.5296	1.5210	1.5258	1.5168
PF_2HSe (**10**)	P-Se	2.0016	2.0052	2.0060	2.0304	2.0160	2.0278	2.0277	2.0318	2.0095
$S(GeH_3)_2$ (**11**)	Ge-S	2.1979	2.2174	2.1949	2.2344	2.2003	2.2282	2.2243	2.2452	2.1961
	Ge-H	1.4999	1.5326	1.4989	1.5319	1.5109	1.5275	1.5179	1.5242	1.5153
$SeBr_2$ (**12**)	Se-Br	2.2752	2.3063	2.2898	2.3279	2.2915	2.3328	2.3249	2.3621	2.2982
$SeCl_2$ (**13**)	Se-Cl	2.1240	2.1461	2.1324	2.1745	2.1415	2.1707	2.1737	2.1994	2.1385
$Se(SiH_3)_2$ (**14**)	Se-Si	2.2534	2.2670	2.2588	2.2949	2.2677	2.2836	2.2871	2.2948	2.2624

Br_2 (**15**)	Br-Br	2.2545	2.2865	2.2703	2.3139	2.2720	2.3078	2.3040	2.3459	2.2799
Me_2GeF_2 (**16**)	Ge-F	1.7229	1.7424	1.7260	1.7549	1.7242	1.7402	1.7454	1.7690	1.7238
	Ge-C	1.8972	1.9272	1.8960	1.9260	1.9013	1.9266	1.9184	1.9259	1.9027
AsP_3 (**17**)	As-P	2.2936	2.3277	2.2985	2.3447	2.3071	2.3220	2.3270	2.3434	2.2954
HCBrClF (**18**)	Br-C	1.9089	1.9277	1.9121	1.9407	1.9167	1.9435	1.9324	1.9541	1.9197

Table S2. Equilibrium bond lengths (in Å) in molecules **1-18** calculated at the DFT(B97-2) level of theory with different basis sets.

Molecule	Bond	cc-pV5Z	pecG-1	pecG-2	cc-pVDZ	cc-pVTZ	6-31G(d,p)	6-311G(d,p)	pc-1	pc-2
As_4 (**1**)	As-As	2.4259	2.4287	2.4261	2.4358	2.4279	2.4288	2.4355	2.4562	2.4289
$AsBr_3$ (**2**)	As-Br	2.3425	2.3341	2.3420	2.3523	2.3445	2.3467	2.3547	2.3728	2.3472
$AsCl_3$ (**3**)	As-Cl	2.1736	2.1672	2.1725	2.1903	2.1775	2.1872	2.1901	2.2070	2.1777
AsF_3 (**4**)	As-F	1.7155	1.7364	1.7172	1.7445	1.7175	1.7237	1.7411	1.7610	1.7190
$CHBr_3$ (**5**)	C-Br	1.9274	1.9314	1.9276	1.9355	1.9286	1.9321	1.9349	1.9408	1.9295
$GaBr_3$ (**6**)	Ga-Br	2.2668	2.2570	2.2670	2.2783	2.2706	2.2771	2.2714	2.2865	2.2688
$GaCl_3$ (**7**)	Ga-Cl	2.1140	2.1077	2.1135	2.1310	2.1192	2.1298	2.1187	2.1348	2.1147
$GeBr_2$ (**8**)	Ge-Br	2.3494	2.3367	2.3487	2.3591	2.3518	2.3538	2.3581	2.3762	2.3536
$O(GeH_3)_2$ (**9**)	Ge-O	1.7709	1.7868	1.7714	1.7955	1.7727	1.7935	1.7815	1.8064	1.7742
	Ge-H	1.5349	1.5367	1.5353	1.5418	1.5363	1.5360	1.5358	1.5354	1.5359
PF_2HSe (**10**)	P-Se	2.0228	2.0112	2.0212	2.0331	2.0296	2.0255	2.0274	2.0285	2.0231
$S(GeH_3)_2$ (**11**)	Ge-S	2.2258	2.2287	2.2253	2.2402	2.2297	2.2333	2.2375	2.2466	2.2283
	Ge-H	1.5320	1.5336	1.5328	1.5392	1.5335	1.5331	1.5327	1.5334	1.5331
$SeBr_2$ (**12**)	Se-Br	2.3086	2.3091	2.3088	2.3248	2.3115	2.3223	2.3258	2.3472	2.3153
$SeCl_2$ (**13**)	Se-Cl	2.1494	2.1500	2.1492	2.1728	2.1552	2.1707	2.1748	2.1900	2.1555
$Se(SiH_3)_2$ (**14**)	Se-Si	2.2840	2.2798	2.2840	2.3015	2.2898	2.2863	2.2934	2.2960	2.2850
Br_2 (**15**)	Br-Br	2.2834	2.2839	2.2834	2.3049	2.2857	2.2930	2.3040	2.3275	2.2909
Me_2GeF_2 (**16**)	Ge-F	1.7352	1.7481	1.7364	1.7585	1.7366	1.7424	1.7515	1.7695	1.7378
	Ge-C	1.9311	1.9361	1.9300	1.9328	1.9319	1.9342	1.9313	1.9346	1.9322
AsP_3 (**17**)	As-P	2.3005	2.3063	2.3009	2.3191	2.3057	2.3056	2.3091	2.3199	2.2991
HCBrClF (**18**)	Br-C	1.9394	1.9422	1.9403	1.9466	1.9414	1.9452	1.9447	1.9529	1.9423

Table S3. Equilibrium valence bond angles (in arc degrees, °) in molecules **1-18** calculated at the MP2 level of theory with different basis sets taking into account vibrational and relativistic corrections.

Mol.	Angle	cc-pV5Z	pecG-1	pecG-2	cc-pVDZ	cc-pVTZ	6-31G(d,p)	6-311G(d,p)	pc-1	pc-2
$AsBr_3$	BrAsBr	99.23	100.51	99.54	100.16	99.53	99.90	100.12	99.79	99.72
$AsCl_3$	ClAsCl	98.52	99.13	98.73	99.30	98.83	99.03	99.05	98.85	99.17
AsF_3	FAsF	95.95	96.27	95.74	96.37	96.10	96.19	96.16	96.36	95.85
$CHBr_3$	HCBr	107.38	106.86	107.05	107.11	107.27	107.27	106.95	107.30	107.24
$GeBr_2$	BrGeBr	100.05	101.88	100.85	101.49	100.84	101.40	101.23	101.43	100.76
$O(GeH_3)_2$	GeOGe	126.2	126.3	129.32	127.29	129.11	126.23	127.40	126.87	128.51
PF_2HSe	SePF	117.43	118.09	117.44	117.80	117.52	117.49	117.42	117.69	117.46
$S(GeH_3)_2$	GeSGe	96.19	97.36	96.23	98.13	96.47	97.67	98.24	97.32	97.16
$SeBr_2$	BrSeBr	101.06	101.90	101.53	101.78	101.45	101.49	102.01	101.27	101.56
$SeCl_2$	ClSeCl	100.33	100.48	100.62	100.94	100.69	100.63	100.79	100.38	100.99
$Se(SiH_3)_2$	SiSeSi	93.98	95.36	94.10	95.59	93.98	97.74	96.12	97.45	95.36
HCBrClF	FCBr	109.24	109.23	109.30	109.37	109.43	108.77	109.16	109.00	109.30

Table S4. Equilibrium valence bond angles (in arc degrees, °) in molecules **1-18** calculated at the DFT(B97-2) level of theory with different basis sets taking into account vibrational and relativistic corrections.

Mol.	Angle	cc-pV5Z	pecG-1	pecG-2	cc-pVDZ	cc-pVTZ	6-31G(d,p)	6-311G(d,p)	pc-1	pc-2
$AsBr_3$	BrAsBr	100.67	101.17	100.71	100.80	100.71	100.56	100.84	100.50	100.73
$AsCl_3$	ClAsCl	99.59	99.71	99.55	99.70	99.62	99.44	99.53	99.30	99.69
AsF_3	FAsF	96.16	96.37	96.03	96.37	96.19	96.20	96.23	96.18	96.06
$CHBr_3$	HCBr	106.90	106.77	106.85	106.92	106.89	107.03	106.73	107.05	106.91
$GeBr_2$	BrGeBr	101.73	102.30	101.78	101.88	101.76	101.70	101.95	101.75	101.76
$O(GeH_3)_2$	GeOGe	129.43	127.94	129.41	128.11	129.61	127.50	130.14	127.93	129.37
PF_2HSe	SePF	117.42	117.82	117.44	117.61	117.45	117.44	117.45	117.63	117.46
$S(GeH_3)_2$	GeSGe	100.34	100.16	100.38	100.40	100.39	100.20	100.27	100.38	100.40
$SeBr_2$	BrSeBr	103.24	103.63	103.28	103.31	103.26	103.03	103.39	102.93	103.26
$SeCl_2$	ClSeCl	101.95	102.00	101.94	102.09	102.00	101.67	101.80	101.46	102.03
$Se(SiH_3)_2$	SiSeSi	96.99	97.12	96.91	96.98	96.89	97.70	96.85	97.68	96.98
HCBrClF	FCBr	109.32	109.19	109.28	109.36	109.37	109.23	109.26	109.21	109.37

Table S5. Total bond lengths (in Å) in molecules **1-18** calculated at the CCSD(T) level of theory with the pecG-1 and pecG-2 basis sets taking into account vibrational and relativistic corrections, against experiment.[a]

Molecule	Bond	Equilibrium Bond length (r_e)	Equilibrium Bond length (r_e)	Vibrational correction (Δr_{vib})	Relativistic correction Δr_{rel}	Total (r_e + Δr_{vib} + Δr_{rel}) with r_e (**pecG-1**)	Total (r_e + Δr_{vib} + Δr_{rel}) with r_e (**pecG-2**)	Exp.
		**pecG-1**	**pecG-2**	pecG-1	dyall.v3z			
As_4 (**1**)	As-As	2.4573	2.4235	0.0035	-0.0050	2.4558	2.4220	2.435 ± 0.004
$AsBr_3$ (**2**)	As-Br	2.3367	2.3256	0.0026	-0.0030	2.3363	2.3252	2.3244 ± 0.0024
$AsCl_3$ (**3**)	As-Cl	2.1658	2.1556	0.0042	0.0010	2.1710	2.1608	2.1621 ± 0.0033
AsF_3 (**4**)	As-F	1.7307	1.7033	0.0045	0.0023	1.7375	1.7101	1.7089 ± 0.0016
$CHBr_3$ (**5**)	C-Br	1.9378	1.9202	0.0067	-0.0012	1.9433	1.9257	1.924 ± 0.005
$GaBr_3$ (**6**)	Ga-Br	2.2567	2.2403	0.0030	-0.0074	2.2523	2.2359	2.239 ± 0.007
$GaCl_3$ (**7**)	Ga-Cl	2.1099	2.0955	0.0021	-0.0048	2.1072	2.0928	2.108 ± 0.003
$GeBr_2$ (**8**)	Ge-Br	2.3308	2.3227	0.0031	-0.0045	2.3294	2.3213	2.337 ± 0.013
$O(GeH_3)_2$ (**9**)	Ge-O	1.7841	1.7550	0.0051	0.0009	1.7901	1.7610	1.766 ± 0.004
	Ge-H	1.5418	1.5104	0.0206	-0.0070	1.5554	1.5240	1.531 ± 0.011
PF_2HSe (**10**)	P-Se	2.0166	2.0193	0.0036	-0.0021	2.0181	2.0208	2.026 ± 0.004
$S(GeH_3)_2$ (**11**)	Ge-S	2.2289	2.2077	0.0062	-0.0029	2.2322	2.2110	2.209 ± 0.004
	Ge-H	1.5395	1.5085	0.0205	-0.0066	1.5534	1.5224	1.512 ± 0.016
$SeBr_2$ (**12**)	Se-Br	2.3281	2.3103	0.0033	-0.0017	2.3297	2.3119	2.306 ± 0.005
$SeCl_2$ (**13**)	Se-Cl	2.1651	2.1499	0.0035	0.0011	2.1697	2.1545	2.157 ± 0.003
$Se(SiH_3)_2$ (**14**)	Se-Si	2.2778	2.2696	0.0070	-0.0027	2.2821	2.2739	2.2730 ± 0.0035
Br_2 (**15**)	Br-Br	2.3112	2.2943	0.0031	-0.0006	2.3137	2.2968	2.2873
Me_2GeF_2 (**16**)	Ge-C	1.9346	1.9049	0.0064	-0.0051	1.9359	1.9062	1.928 ± 0.003
	Ge-F	1.7422	1.7249	0.0043	-0.0003	1.7462	1.7289	1.739 ± 0.002
AsP_3 (**17**)	As-P	2.3419	2.3088	0.0046	-0.0012	2.3453	2.3122	2.3041 ± 0.0012
HCBrClF (**18**)	Br-C	1.9383	1.9240	0.0053	-0.0009	1.9427	1.9284	1.927 ± 0.006

[a] Experimental values are taken from different sources referenced in the main text, see references [54-69].

Table S6. Total bond lengths (in Å) in molecules **1-18** calculated at the CCSD(T) level of theory with the cc-pVDZ and cc-pVTZ basis sets taking into account vibrational and relativistic corrections, against experiment.[a]

Molecule	Bond	Equilibrium Bond length (r_e)	Equilibrium Bond length (r_e)	Vibrational correction (Δr_{vib})	Relativistic correction Δr_{rel}	Total (r_e + Δr_{vib} + Δr_{rel}) with r_e (cc-pVDZ)	Total (r_e + Δr_{vib} + Δr_{rel}) with r_e (cc-pVTZ)	Exp.
		cc-pVDZ	cc-pVTZ	cc-pVDZ	dyall.v3z			
As_4 (**1**)	As-As	2.4689	2.4294	0.0035	-0.0050	2.4674	2.4279	2.435 ± 0.004

$AsBr_3$ (**2**)	As-Br	2.3610	2.3290	0.0027	-0.0030	2.3607	2.3287	2.3244 ± 0.0024
$AsCl_3$ (**3**)	As-Cl	2.1942	2.1649	0.0044	0.0010	2.1996	2.1703	2.1621 ± 0.0033
AsF_3 (**4**)	As-F	1.7418	1.7022	0.0045	0.0023	1.7486	1.7090	1.7089 ± 0.0016
$CHBr_3$ (**5**)	C-Br	1.9474	1.9238	0.0073	-0.0012	1.9535	1.9299	1.924 ± 0.005
$GaBr_3$ (**6**)	Ga-Br	2.2813	2.2436	0.0030	-0.0074	2.2769	2.2392	2.239 ± 0.007
$GaCl_3$ (**7**)	Ga-Cl	2.1352	2.1040	0.0022	-0.0048	2.1326	2.1014	2.108 ± 0.003
$GeBr_2$ (**8**)	Ge-Br	2.3583	2.3257	0.0031	-0.0045	2.3569	2.3243	2.337 ± 0.013
$O(GeH_3)_2$ (**9**)	Ge-O	1.7960	1.7581	0.0055	0.0009	1.8024	1.7645	1.766 ± 0.004
	Ge-H	1.5393	1.5213	0.0206	-0.0070	1.5529	1.5349	1.531 ± 0.011
PF_2HSe (**10**)	P-Se	2.0434	2.0296	0.0038	-0.0021	2.0451	2.0313	2.026 ± 0.004
$S(GeH_3)_2$ (**11**)	Ge-S	2.2453	2.2127	0.0063	-0.0029	2.2487	2.2161	2.209 ± 0.004
	Ge-H	1.5375	1.5197	0.0205	-0.0066	1.5514	1.5336	1.512 ± 0.016
$SeBr_2$ (**12**)	Se-Br	2.3499	2.3128	0.0034	-0.0017	2.3516	2.3145	2.306 ± 0.005
$SeCl_2$ (**13**)	Se-Cl	2.1938	2.1590	0.0037	0.0011	2.1986	2.1638	2.157 ± 0.003
$Se(SiH_3)_2$ (**14**)	Se-Si	2.3057	2.2785	0.0066	-0.0027	2.3096	2.2824	2.2730 ± 0.0035
Br_2 (**15**)	Br-Br	2.3406	2.2962	0.0032	-0.0006	2.3432	2.2988	2.2873
Me_2GeF_2 (**16**)	Ge-C	1.9331	1.9094	0.0069	-0.0051	1.9349	1.9112	1.928 ± 0.003
	Ge-F	1.7555	1.7230	0.0043	-0.0003	1.7595	1.7270	1.739 ± 0.002
AsP_3 (**17**)	As-P	2.3453	2.3163	0.0046	-0.0012	2.3487	2.3197	2.3041 ± 0.0012
HCBrClF (**18**)	Br-C	1.9510	1.9289	0.0054	-0.0009	1.9555	1.9334	1.927 ± 0.006

[a] Experimental values are taken from different sources referenced in the main text, see references [54-69].

Table S7. Total bond lengths (in Å) in molecules **1-18** calculated at the CCSD(T) level of theory with the 6-31G(d,p) and 6-311G(d,p) basis sets taking into account vibrational and relativistic corrections, against experiment.[a]

Molecule	Bond	Equilibrium Bond length (r_e)	Equilibrium Bond length (r_e)	Vibrational correction (Δr_{vib})	Relativistic correction Δr_{rel}	Total (r_e + Δr_{vib} + Δr_{rel}) with r_e (6-31G(d,p))	Total (r_e + Δr_{vib} + Δr_{rel}) with r_e (6-311G(d,p))	Exp.
		6-31G(d,p)	6-311G(d,p)	6-31G(d,p)	dyall.v3z			
As_4 (**1**)	As-As	2.4610	2.4740	0.0036	-0.0050	2.4596	2.4726	2.435 ± 0.004
$AsBr_3$ (**2**)	As-Br	2.3546	2.3616	0.0027	-0.0030	2.3543	2.3613	2.3244 ± 0.0024
$AsCl_3$ (**3**)	As-Cl	2.1862	2.1922	0.0045	0.0010	2.1917	2.1977	2.1621 ± 0.0033
AsF_3 (**4**)	As-F	1.7234	1.7354	0.0044	0.0023	1.7301	1.7421	1.7089 ± 0.0016
$CHBr_3$ (**5**)	C-Br	1.9407	1.9408	0.0072	-0.0012	1.9467	1.9468	1.924 ± 0.005
$GaBr_3$ (**6**)	Ga-Br	2.2718	2.2712	0.0031	-0.0074	2.2675	2.2669	2.239 ± 0.007
$GaCl_3$ (**7**)	Ga-Cl	2.1202	2.1170	0.0023	-0.0048	2.1177	2.1145	2.108 ± 0.003
$GeBr_2$ (**8**)	Ge-Br	2.3489	2.3567	0.0032	-0.0045	2.3476	2.3554	2.337 ± 0.013
$O(GeH_3)_2$ (**9**)	Ge-O	1.7909	1.7816	0.0054	0.0009	1.7972	1.7879	1.766 ± 0.004

	Ge-H	1.5326	1.5287	0.0206	-0.0070	1.5462	1.5423	1.531 ± 0.011
PF_2HSe (**10**)	P-Se	2.0398	2.0423	0.0037	-0.0021	2.0414	2.0439	2.026 ± 0.004
$S(GeH_3)_2$ (**11**)	Ge-S	2.2303	2.2378	0.0061	-0.0029	2.2335	2.241	2.209 ± 0.004
	Ge-H	1.5301	1.5259	0.0206	-0.0066	1.5441	1.5399	1.512 ± 0.016
$SeBr_2$ (**12**)	Se-Br	2.3413	2.3473	0.0033	-0.0017	2.3429	2.3489	2.306 ± 0.005
$SeCl_2$ (**13**)	Se-Cl	2.1896	2.1933	0.0036	0.0011	2.1943	2.198	2.157 ± 0.003
$Se(SiH_3)_2$ (**14**)	Se-Si	2.2904	2.2983	0.0066	-0.0027	2.2943	2.3022	2.2730 ± 0.0035
Br_2 (**15**)	Br-Br	2.3286	2.3292	0.0032	-0.0006	2.3312	2.3318	2.2873
Me_2GeF_2 (**16**)	Ge-C	1.9272	1.9269	0.0069	-0.0051	1.929	1.9287	1.928 ± 0.003
	Ge-F	1.7407	1.7452	0.0040	-0.0003	1.7444	1.7489	1.739 ± 0.002
AsP_3 (**17**)	As-P	2.3327	2.3413	0.0046	-0.0012	2.3361	2.3447	2.3041 ± 0.0012
HCBrClF (**18**)	Br-C	1.9445	1.9433	0.0052	-0.0009	1.9488	1.9476	1.927 ± 0.006

[a] Experimental values are taken from different sources referenced in the main text, see references [54-69].

Table S8. Total bond lengths (in Å) in molecules **1-18** calculated at the CCSD(T) level of theory with the pc-1 and pc-2 basis sets taking into account vibrational and relativistic corrections, against experiment.[a]

Molecule	Bond	Equilibrium Bond length (r_e)	Equilibrium Bond length (r_e)	Vibrational correction (Δr_{vib})	Relativistic correction Δr_{rel}	Total (r_e + Δr_{vib} + Δr_{rel}) with r_e (pc-1)	Total (r_e + Δr_{vib} + Δr_{rel}) with r_e (pc-2)	Exp.
		pc-1	pc-2	pc-1	dyall.v3z			
As_4 (**1**)	As-As	2.5055	2.4318	0.0034	-0.0050	2.5039	2.4302	2.435 ± 0.004
$AsBr_3$ (**2**)	As-Br	2.3925	2.3333	0.0025	-0.0030	2.3920	2.3328	2.3244 ± 0.0024
$AsCl_3$ (**3**)	As-Cl	2.2189	2.1590	0.0045	0.0010	2.2244	2.1645	2.1621 ± 0.0033
AsF_3 (**4**)	As-F	1.7612	1.7016	0.0047	0.0023	1.7682	1.7086	1.7089 ± 0.0016
$CHBr_3$ (**5**)	C-Br	1.9582	1.9259	0.0072	-0.0012	1.9642	1.9319	1.924 ± 0.005
$GaBr_3$ (**6**)	Ga-Br	2.2944	2.2485	0.0031	-0.0074	2.2901	2.2442	2.239 ± 0.007
$GaCl_3$ (**7**)	Ga-Cl	2.1403	2.0986	0.0023	-0.0048	2.1378	2.0961	2.108 ± 0.003
$GeBr_2$ (**8**)	Ge-Br	2.3880	2.3301	0.0032	-0.0045	2.3867	2.3288	2.337 ± 0.013
$O(GeH_3)_2$ (**9**)	Ge-O	1.8079	1.7620	0.0058	0.0009	1.8146	1.7687	1.766 ± 0.004
	Ge-H	1.5316	1.5244	0.0217	-0.0070	1.5463	1.5391	1.531 ± 0.011
PF_2HSe (**10**)	P-Se	2.0440	2.0228	0.0038	-0.0021	2.0457	2.0245	2.026 ± 0.004
$S(GeH_3)_2$ (**11**)	Ge-S	2.2568	2.2076	0.0061	-0.0029	2.2600	2.2108	2.209 ± 0.004
	Ge-H	1.5303	1.5228	0.0206	-0.0066	1.5443	1.5368	1.512 ± 0.016
$SeBr_2$ (**12**)	Se-Br	2.3825	2.3199	0.0032	-0.0017	2.3840	2.3214	2.306 ± 0.005
$SeCl_2$ (**13**)	Se-Cl	2.2189	2.1560	0.0037	0.0011	2.2237	2.1608	2.157 ± 0.003
$Se(SiH_3)_2$ (**14**)	Se-Si	2.3032	2.2725	0.0065	-0.0027	2.3070	2.2763	2.2730 ± 0.0035
Br_2 (**15**)	Br-Br	2.3709	2.3049	0.0032	-0.0006	2.3735	2.3075	2.2873

Me_2GeF_2 (**16**)	Ge-C	1.9327	1.9103	0.0069	-0.0051	1.9345	1.9121	1.928 ± 0.003
	Ge-F	1.7679	1.7223	0.0040	-0.0003	1.7716	1.7260	1.739 ± 0.002
AsP_3 (**17**)	As-P	2.3573	2.3070	0.0046	-0.0012	2.3607	2.3104	2.3041 ± 0.0012
HCBrClF (**18**)	Br-C	1.9640	1.9325	0.0054	-0.0009	1.9685	1.9370	1.927 ± 0.006

[a] Experimental values are taken from different sources referenced in the main text, see references [54-69].

Table S9. Total bond lengths (in Å) in molecules **1-18** calculated at the DFT(B97-2) level of theory with the pecG-1 and pecG-2 basis sets taking into account vibrational and relativistic corrections, against experiment.[a]

Molecule	Bond	Equilibrium Bond length (r_e)	Equilibrium Bond length (r_e)	Vibrational correction (Δr_{vib})	Relativistic correction Δr_{rel}	Total (r_e + Δr_{vib} + Δr_{rel}) with r_e	Total (r_e + Δr_{vib} + Δr_{rel}) with r_e	Exp.
		**pecG-1**	**pecG-2**	pecG-1	dyall.v3z	(**pecG-1**)	(**pecG-2**)	
As_4 (**1**)	As-As	2.4287	2.4261	0.0035	-0.0050	2.4272	2.4246	2.435 ± 0.004
$AsBr_3$ (**2**)	As-Br	2.3341	2.3420	0.0026	-0.0030	2.3337	2.3416	2.3244 ± 0.0024
$AsCl_3$ (**3**)	As-Cl	2.1672	2.1725	0.0042	0.0010	2.1724	2.1777	2.1621 ± 0.0033
AsF_3 (**4**)	As-F	1.7364	1.7172	0.0045	0.0023	1.7432	1.7240	1.7089 ± 0.0016
$CHBr_3$ (**5**)	C-Br	1.9314	1.9276	0.0067	-0.0012	1.9369	1.9331	1.924 ± 0.005
$GaBr_3$ (**6**)	Ga-Br	2.2570	2.2670	0.0030	-0.0074	2.2526	2.2626	2.239 ± 0.007
$GaCl_3$ (**7**)	Ga-Cl	2.1077	2.1135	0.0021	-0.0048	2.1050	2.1108	2.108 ± 0.003
$GeBr_2$ (**8**)	Ge-Br	2.3367	2.3487	0.0031	-0.0045	2.3353	2.3473	2.337 ± 0.013
$O(GeH_3)_2$ (**9**)	Ge-O	1.7868	1.7714	0.0051	0.0009	1.7928	1.7774	1.766 ± 0.004
	Ge-H	1.5367	1.5353	0.0206	-0.0070	1.5503	1.5489	1.531 ± 0.011
PF_2HSe (**10**)	P-Se	2.0112	2.0212	0.0036	-0.0021	2.0127	2.0227	2.026 ± 0.004
$S(GeH_3)_2$ (**11**)	Ge-S	2.2287	2.2253	0.0062	-0.0029	2.2320	2.2286	2.209 ± 0.004
	Ge-H	1.5336	1.5328	0.0205	-0.0066	1.5475	1.5467	1.512 ± 0.016
$SeBr_2$ (**12**)	Se-Br	2.3091	2.3088	0.0033	-0.0017	2.3107	2.3104	2.306 ± 0.005
$SeCl_2$ (**13**)	Se-Cl	2.1500	2.1492	0.0035	0.0011	2.1546	2.1538	2.157 ± 0.003
$Se(SiH_3)_2$ (**14**)	Se-Si	2.2798	2.2840	0.0070	-0.0027	2.2841	2.2883	2.2730 ± 0.0035
Br_2 (**15**)	Br-Br	2.2839	2.2834	0.0031	-0.0006	2.2864	2.2859	2.2873
Me_2GeF_2 (**16**)	Ge-F	1.7481	1.7364	0.0064	-0.0003	1.7542	1.7425	1.928 ± 0.003
	Ge-C	1.9361	1.9300	0.0043	-0.0051	1.9353	1.9292	1.739 ± 0.002
AsP_3 (**17**)	As-P	2.3063	2.3009	0.0046	-0.0012	2.3097	2.3043	2.3041 ± 0.0012
HCBrClF (**18**)	Br-C	1.9422	1.9403	0.0053	-0.0009	1.9466	1.9447	1.927 ± 0.006

[a] Experimental values are taken from different sources referenced in the main text, see references [54-69].

Table S10. Total bond lengths (in Å) in molecules **1-18** calculated at the DFT(B97-2) level of theory with the cc-pVDZ and cc-pVTZ basis sets taking into account vibrational and relativistic corrections, against experiment.[a]

Molecule	Bond	Equilibrium Bond length (r_e)	Equilibrium Bond length (r_e)	Vibrational correction (Δr_{vib})	Relativistic correction Δr_{rel}	Total ($r_e + \Delta r_{vib} + \Delta r_{rel}$) with r_e (cc-pVDZ)	Total ($r_e + \Delta r_{vib} + \Delta r_{rel}$) with r_e (cc-pVTZ)	Exp.
		cc-pVDZ	cc-pVTZ	cc-pVDZ	dyall.v3z			
As_4 (**1**)	As-As	2.4358	2.4279	0.0035	-0.0050	2.4343	2.4264	2.435 ± 0.004
$AsBr_3$ (**2**)	As-Br	2.3523	2.3445	0.0027	-0.0030	2.3520	2.3442	2.3244 ± 0.0024
$AsCl_3$ (**3**)	As-Cl	2.1903	2.1775	0.0044	0.0010	2.1957	2.1829	2.1621 ± 0.0033
AsF_3 (**4**)	As-F	1.7445	1.7175	0.0045	0.0023	1.7513	1.7243	1.7089 ± 0.0016
$CHBr_3$ (**5**)	C-Br	1.9355	1.9286	0.0073	-0.0012	1.9416	1.9347	1.924 ± 0.005
$GaBr_3$ (**6**)	Ga-Br	2.2783	2.2706	0.0030	-0.0074	2.2739	2.2662	2.239 ± 0.007
$GaCl_3$ (**7**)	Ga-Cl	2.1310	2.1192	0.0022	-0.0048	2.1284	2.1166	2.108 ± 0.003
$GeBr_2$ (**8**)	Ge-Br	2.3591	2.3518	0.0031	-0.0045	2.3577	2.3504	2.337 ± 0.013
$O(GeH_3)_2$ (**9**)	Ge-O	1.7955	1.7727	0.0055	0.0009	1.8019	1.7791	1.766 ± 0.004
	Ge-H	1.5418	1.5363	0.0206	-0.0070	1.5554	1.5499	1.531 ± 0.011
PF_2HSe (**10**)	P-Se	2.0331	2.0296	0.0038	-0.0021	2.0348	2.0313	2.026 ± 0.004
$S(GeH_3)_2$ (**11**)	Ge-S	2.2402	2.2297	0.0063	-0.0029	2.2436	2.2331	2.209 ± 0.004
	Ge-H	1.5392	1.5335	0.0205	-0.0066	1.5531	1.5474	1.512 ± 0.016
$SeBr_2$ (**12**)	Se-Br	2.3248	2.3115	0.0034	-0.0017	2.3265	2.3132	2.306 ± 0.005
$SeCl_2$ (**13**)	Se-Cl	2.1728	2.1552	0.0037	0.0011	2.1776	2.1600	2.157 ± 0.003
$Se(SiH_3)_2$ (**14**)	Se-Si	2.3015	2.2898	0.0066	-0.0027	2.3054	2.2937	2.2730 ± 0.0035
Br_2 (**15**)	Br-Br	2.3049	2.2857	0.0032	-0.0006	2.3075	2.2883	2.2873
Me_2GeF_2 (**16**)	Ge-F	1.7585	1.7366	0.0069	-0.0003	1.7651	1.7432	1.928 ± 0.003
	Ge-C	1.9328	1.9319	0.0043	-0.0051	1.9320	1.9311	1.739 ± 0.002
AsP_3 (**17**)	As-P	2.3191	2.3057	0.0046	-0.0012	2.3225	2.3091	2.3041 ± 0.0012
HCBrClF (**18**)	Br-C	1.9466	1.9414	0.0054	-0.0009	1.9511	1.9459	1.927 ± 0.006

[a] Experimental values are taken from different sources referenced in the main text, see references [54-69].

Table S11. Total bond lengths (in Å) in molecules **1-18** calculated at the DFT(B97-2) level of theory with the 6-31G(d,p) and 6-311G(d,p) basis sets taking into account vibrational and relativistic corrections, against experiment.[a]

Molecule	Bond	Equilibrium Bond length (r_e)	Equilibrium Bond length (r_e)	Vibrational correction (Δr_{vib})	Relativistic correction Δr_{rel}	Total ($r_e + \Delta r_{vib} + \Delta r_{rel}$) with r_e	Total ($r_e + \Delta r_{vib} + \Delta r_{rel}$) with r_e	Exp.

		6-31G(d,p)	6-311G(d,p)	6-31G(d,p)	dyall.v3z	(6-31G(d,p))	(6-311G(d,p)	
As_4 (**1**)	As-As	2.4288	2.4355	0.0036	-0.0050	2.4274	2.4341	2.435 ± 0.004
$AsBr_3$ (**2**)	As-Br	2.3467	2.3547	0.0027	-0.0030	2.3464	2.3544	2.3244 ± 0.0024
$AsCl_3$ (**3**)	As-Cl	2.1872	2.1901	0.0045	0.0010	2.1927	2.1956	2.1621 ± 0.0033
AsF_3 (**4**)	As-F	1.7237	1.7411	0.0044	0.0023	1.7304	1.7478	1.7089 ± 0.0016
$CHBr_3$ (**5**)	C-Br	1.9321	1.9349	0.0072	-0.0012	1.9381	1.9409	1.924 ± 0.005
$GaBr_3$ (**6**)	Ga-Br	2.2771	2.2714	0.0031	-0.0074	2.2728	2.2671	2.239 ± 0.007
$GaCl_3$ (**7**)	Ga-Cl	2.1298	2.1187	0.0023	-0.0048	2.1273	2.1162	2.108 ± 0.003
$GeBr_2$ (**8**)	Ge-Br	2.3538	2.3581	0.0032	-0.0045	2.3525	2.3568	2.337 ± 0.013
$O(GeH_3)_2$ (**9**)	Ge-O	1.7935	1.7815	0.0054	0.0009	1.7998	1.7878	1.766 ± 0.004
	Ge-H	1.5360	1.5358	0.0206	-0.0070	1.5496	1.5494	1.531 ± 0.011
PF_2HSe (**10**)	P-Se	2.0255	2.0274	0.0037	-0.0021	2.0271	2.0290	2.026 ± 0.004
$S(GeH_3)_2$ (**11**)	Ge-S	2.2333	2.2375	0.0061	-0.0029	2.2365	2.2407	2.209 ± 0.004
	Ge-H	1.5331	1.5327	0.0206	-0.0066	1.5471	1.5467	1.512 ± 0.016
$SeBr_2$ (**12**)	Se-Br	2.3223	2.3258	0.0033	-0.0017	2.3239	2.3274	2.306 ± 0.005
$SeCl_2$ (**13**)	Se-Cl	2.1707	2.1748	0.0036	0.0011	2.1754	2.1795	2.157 ± 0.003
$Se(SiH_3)_2$ (**14**)	Se-Si	2.2863	2.2934	0.0066	-0.0027	2.2902	2.2973	2.2730 ± 0.0035
Br_2 (**15**)	Br-Br	2.2930	2.3040	0.0032	-0.0006	2.2956	2.3066	2.2873
Me_2GeF_2 (**16**)	Ge-F	1.7424	1.7515	0.0069	-0.0003	1.7490	1.7581	1.928 ± 0.003
	Ge-C	1.9342	1.9313	0.0040	-0.0051	1.9331	1.9302	1.739 ± 0.002
AsP_3 (**17**)	As-P	2.3056	2.3091	0.0046	-0.0012	2.3090	2.3125	2.3041 ± 0.0012
HCBrClF (**18**)	Br-C	1.9452	1.9447	0.0052	-0.0009	1.9495	1.9490	1.927 ± 0.006

[a] Experimental values are taken from different sources referenced in the main text, see references [54-69].

Table S12. Total bond lengths (in Å) in molecules **1-18** calculated at the DFT(B97-2) level of theory with the pc-1 and pc-2 basis sets taking into account vibrational and relativistic corrections, against experiment.[a]

Molecule	Bond	Equilibrium Bond length (r_e)	Equilibrium Bond length (r_e)	Vibrational correction (Δr_{vib})	Relativistic correction Δr_{rel}	Total (r_e + Δr_{vib} + Δr_{rel}) with r_e (pc-1)	Total (r_e + Δr_{vib} + Δr_{rel}) with r_e (pc-2)	Exp.
		pc-1	pc-2	pc-1	dyall.v3z			
As_4 (**1**)	As-As	2.4562	2.4289	0.0034	-0.0050	2.4546	2.4273	2.435 ± 0.004
$AsBr_3$ (**2**)	As-Br	2.3728	2.3472	0.0025	-0.0030	2.3723	2.3467	2.3244 ± 0.0024
$AsCl_3$ (**3**)	As-Cl	2.2070	2.1777	0.0045	0.0010	2.2125	2.1832	2.1621 ± 0.0033
AsF_3 (**4**)	As-F	1.7610	1.7190	0.0047	0.0023	1.7680	1.7260	1.7089 ± 0.0016
$CHBr_3$ (**5**)	C-Br	1.9408	1.9295	0.0072	-0.0012	1.9468	1.9355	1.924 ± 0.005
$GaBr_3$ (**6**)	Ga-Br	2.2865	2.2688	0.0031	-0.0074	2.2822	2.2645	2.239 ± 0.007
$GaCl_3$ (**7**)	Ga-Cl	2.1348	2.1147	0.0023	-0.0048	2.1323	2.1122	2.108 ± 0.003

$GeBr_2$ (**8**)	Ge-Br	2.3762	2.3536	0.0032	-0.0045	2.3749	2.3523	2.337 ± 0.013
$O(GeH_3)_2$ (**9**)	Ge-O	1.8064	1.7742	0.0058	0.0009	1.8131	1.7809	1.766 ± 0.004
	Ge-H	1.5354	1.5359	0.0217	-0.0070	1.5501	1.5506	1.531 ± 0.011
PF_2HSe (**10**)	P-Se	2.0285	2.0231	0.0038	-0.0021	2.0302	2.0248	2.026 ± 0.004
$S(GeH_3)_2$ (**11**)	Ge-S	2.2466	2.2283	0.0061	-0.0029	2.2498	2.2315	2.209 ± 0.004
	Ge-H	1.5334	1.5331	0.0206	-0.0066	1.5474	1.5471	1.512 ± 0.016
$SeBr_2$ (**12**)	Se-Br	2.3472	2.3153	0.0032	-0.0017	2.3487	2.3168	2.306 ± 0.005
$SeCl_2$ (**13**)	Se-Cl	2.1900	2.1555	0.0037	0.0011	2.1948	2.1603	2.157 ± 0.003
$Se(SiH_3)_2$ (**14**)	Se-Si	2.2960	2.2850	0.0065	-0.0027	2.2998	2.2888	2.2730 ± 0.0035
Br_2 (**15**)	Br-Br	2.3275	2.2909	0.0032	-0.0006	2.3301	2.2935	2.2873
Me_2GeF_2 (**16**)	Ge-F	1.7695	1.7378	0.0069	-0.0003	1.7761	1.7444	1.928 ± 0.003
	Ge-C	1.9346	1.9322	0.0040	-0.0051	1.9335	1.9311	1.739 ± 0.002
AsP_3 (**17**)	As-P	2.3199	2.2991	0.0046	-0.0012	2.3233	2.3025	2.3041 ± 0.0012
HCBrClF (**18**)	Br-C	1.9529	1.9423	0.0054	-0.0009	1.9574	1.9468	1.927 ± 0.006

[a] Experimental values are taken from different sources referenced in the main text, see references [54-69].

Table S13. Static dipole polarizability (in au) calculated at the DFT(B97-2)/aug-cc-pVQZ level on different equilibrium geometries obtained within the DFT(B97-2) method with various basis sets, including the pecG-*n* (*n* = 1, 2).

#	Molecule	**Basis sets used in the geometry optimization stage**								
		**cc-pV5Z**	**6-31G(d,p)**	**6-311G(d,p)**	**pc-1**	**pc-2**	**pecG-1**	**pecG-2**	**cc-pVDZ**	**cc-pVTZ**
**1**	As_4	116.93	117.15	117.66	119.25	117.16	117.14	116.94	117.68	117.08
**2**	$AsBr_3$	101.56	101.77	102.38	103.38	101.87	101.25	101.54	102.22	101.69
**3**	$AsCl_3$	75.09	75.72	75.89	76.67	75.32	74.81	75.02	75.94	75.29
**4**	AsF_3	29.83	30.02	30.43	30.92	29.90	30.33	29.86	30.52	29.88
**5**	$CHBr_3$	79.32	79.55	79.75	80.02	79.43	79.56	79.34	79.81	79.38
**6**	$GaBr_3$	93.76	94.41	94.05	95.00	93.89	93.16	93.77	94.48	94.00
**7**	$GaCl_3$	67.84	68.63	68.08	68.87	67.88	67.54	67.82	68.68	68.10
**8**	$GeBr_2$	79.55	79.71	79.90	80.55	79.71	79.17	79.53	79.93	79.65
**9**	$O(GeH_3)_2$	66.55	67.17	66.97	67.50	66.71	67.00	66.60	67.64	66.72
**10**	PF_2HSe	53.49	53.89	53.93	54.14	53.53	53.39	53.45	54.59	53.83
**11**	$S(GeH_3)_2$	86.95	87.26	87.37	87.77	87.11	87.15	86.99	87.94	87.19
**12**	$SeBr_2$	74.69	75.20	75.40	76.18	74.96	74.78	74.71	75.35	74.81
**13**	$SeCl_2$	57.88	58.54	58.69	59.16	58.09	57.91	57.88	58.65	58.08
**14**	$Se(SiH_3)_2$	86.81	87.25	87.41	87.58	86.87	86.85	86.84	88.34	87.26
**15**	Br_2	44.62	44.78	44.97	45.38	44.74	44.62	44.62	44.98	44.66

**16**	Me_2GeF_2	53.60	53.91	53.97	54.57	53.68	53.95	53.59	54.50	53.66
**17**	AsP_3	96.69	97.27	97.49	97.87	96.66	97.06	96.66	98.26	97.16
**18**	HCBrClF	51.42	51.68	51.66	51.81	51.50	51.44	51.43	51.79	51.52
